# Post-transcriptional regulation and subcellular localization of G-protein γ7 subunit: implications for striatal function and behavioral responses to cocaine

**DOI:** 10.3389/fnana.2024.1394659

**Published:** 2024-05-02

**Authors:** Oliver B. Pelletier, Gloria Brunori, Yingcai Wang, Janet D. Robishaw

**Affiliations:** ^1^Department of Biomedical Science, Charles E. Schmidt College of Medicine, Florida Atlantic University, Boca Raton, FL, United States; ^2^Department of Comparative, Diagnostic, and Population Medicine, College of Veterinary Medicine, University of Florida, Gainesville, FL, United States

**Keywords:** G-protein assembly, *Gng7*, 3’UTR, striatum, spatiotemporal control

## Abstract

The striatal D_1_ dopamine receptor (D_1_R) and A_2a_ adenosine receptor (A_2a_R) signaling pathways play important roles in drug-related behaviors. These receptors activate the G_olf_ protein comprised of a specific combination of α_olf_β_2_γ_7_ subunits. During assembly, the γ_7_ subunit sets the cellular level of the G_olf_ protein. In turn, the amount of G_olf_ protein determines the collective output from both D_1_R and A_2a_R signaling pathways. This study shows the *Gng7* gene encodes multiple γ_7_ transcripts differing only in their non-coding regions. In striatum, Transcript 1 is the predominant isoform. Preferentially expressed in the neuropil, Transcript 1 is localized in dendrites where it undergoes post-transcriptional regulation mediated by regulatory elements in its 3′ untranslated region that contribute to translational suppression of the γ_7_ protein. Earlier studies on gene-targeted mice demonstrated loss of γ_7_ protein disrupts assembly of the G_olf_ protein. In the current study, morphological analysis reveals the loss of the G_olf_ protein is associated with altered dendritic morphology of medium spiny neurons. Finally, behavioral analysis of conditional knockout mice with cell-specific deletion of the γ_7_ protein in distinct populations of medium spiny neurons reveals differential roles of the G_olf_ protein in mediating behavioral responses to cocaine. Altogether, these findings provide a better understanding of the regulation of γ_7_ protein expression, its impact on G_olf_ function, and point to a new potential target and mechanisms for treating addiction and related disorders.

## Introduction

The striatum is a key site for the neuroplastic changes that underlie addiction. In the dorsal striatum, psychostimulants increase dopaminergic signaling (Barrot et al., 1999; Bustamante et al., 2002) and evoke the associated cellular changes responsible for the immediate locomotor stimulating response and the later transition to habitual behaviors upon repeated exposure (Nelson and Killcross, 2006; Wickens et al., 2007). In the ventral striatum, these drugs act similarly to increase dopaminergic signaling and induce the cellular adaptations and plasticity leading to addictive behaviors including reinforcement learning, drug seeking, and relapse (Kalivas, 2009; Singer et al., 2016; Furlong et al., 2018). Notably, the neuroanatomic site mediating these actions are the medium spiny neurons (MSNs) that account for >90% of all neurons within these two regions. Further classified based on their projection patterns and receptor expression (Yager et al., 2015), the MSNs expressing the D_1_ dopamine receptor (D_1_R) send projections directly to the basal ganglia output nuclei (i.e., direct circuit), whereas MSNs expressing the D_2_ dopamine receptor (D_2_R) and A_2a_ adenosine receptors (A_2a_R) communicate indirectly to these output nuclei by sending projections through the globus pallidus external and subthalamic nuclei (i.e., indirect circuit) (Yager et al., 2015). In general, the direct circuit acts to initiate behaviors, whereas the indirect circuit serves to inhibit actions (Yager et al., 2015).

Consistent with this spatially encoded circuitry, the D_1_R and A_2a_R signaling pathways expressed in different MSN subpopulations exert distinct roles in drug-related behaviors (Nam et al., 2013; Jones-Tabah et al., 2021). Thus, understanding how these signaling pathways are assembled and regulated is critical for developing therapeutic strategies targeted towards drug use disorders. Within the two distinct MSN populations, both the D_1_R and A_2a_R activate the G_olf_ protein to regulate adenylyl cyclase type 5 (AC5) activity (Gerfen et al., 1990). Our group showed previously that the G_olf_ protein is a specific heterotrimer composed of α_olf,_ β_2,_ γ_7_ subunits, and revealed a critical role for the γ_7_ protein in directing its hierarchical assembly (Schwindinger et al., 2003, 2010). The individual G-αβγ subunits are synthesized as soluble proteins near the rough endoplasmic reticulum (Dupré and Rebois, 2009). There, heterotrimer formation begins when the γ protein interacts with the β protein to form a stable βγ dimer, which subsequently associates with the acylated α protein and the resulting heterotrimer translocates to the plasma membrane (Smrcka, 2008; Dupré and Rebois, 2009; Khan et al., 2013). Our findings support and further extend this model by showing a post-transcriptional requirement for the γ_7_ protein (Schwindinger et al., 2010). By protecting the α_olf_ and β_2_ proteins from degradation, the amount of the γ_7_ protein sets the cellular level of the functional G_olf_ heterotrimer.

Because the amount of the functional G_olf_ heterotrimer represents the rate-limiting step for both the D_1_R and A_2a_R signaling pathways (Belluscio et al., 1998; Hervé et al., 2001; Corvol et al., 2007; Schwindinger et al., 2010), small changes in the γ_7_ protein level could produce consequential changes in Gα_olf_β_2_γ_7_ heterotrimer abundance and signaling functions. However, critical questions remain regarding how the cellular content of γ_7_ protein is regulated and what factor(s) is responsible for ensuring the fidelity of the heterotrimer assembly process. Here, we investigated the gene structure, regional expression, and subcellular localization of the different γ_7_ transcripts. In striatum, we identify a predominant splice variant with a long 3’UTR (>3 kb), containing important regulatory elements for dendritic localization and translational repression of the γ_7_ protein. Furthermore, we reveal a role of the G_olf_β_2_γ_7_ heterotrimer for regulating spine morphology of MSNs. Lastly, we show D_1_R- and A_2a_R-specific deletion of γ_7_ protein differentially influence behavioral responses to cocaine.

## Materials and methods

### Production of *Gng7^−/−^* and Gng7 D_1_R- and D_2_R-conditional knock-out mice

Disruption of *Gng7*, the gene encoding the G-protein γ_7_ subunit in mice, was described previously (Schwindinger et al., 2003). *Gng7^+/−^* heterozygous mice were backcrossed to C57BL/6 mice (Jackson Laboratories, Bar Harbor, ME) for 5 generations. *Gng7^+/−^* mice were intercrossed to produce the *Gng7^−/−^* knockout mice and *Gng7^+/+^* wildtype littermates used in these experiments. To generate *Gng7* D_1_R - and D_2_R - conditional knockout lines, *Gng7*^fl/fl^ mice were bred to either D_1_Cre + (Drd1a; EY262) or D_2_Cre + (Drd2; ER44) for two generations to obtain *Gng7*^fl/fl^ D_1_Cre + or *Gng7*^fl/fl^ D_2_Cre + and *Gng7*^fl/fl^ wildtype littermates, as described previously (Brunori et al., 2021) Mice were genotyped by Transnetyx (Memphis, TN, USA). Animals were group-housed under standard laboratory conditions and kept on a 12 h day/night cycle (lights on at 7:00 A.M.). Male and female mice (8–12 weeks old) were used for all experiments. Mice were maintained in accordance with the National Institutes of Health’s Guide for the care and use of laboratory animals. All methods used were preapproved by the Institutional Animal Care and Use Committee Animals at Florida Atlantic University (Boca Raton, FL).

### AAV *in vitro* infection

HEK293T cells were seeded on each well (0.5 × 10^6 cells) of a 6-well plate in DMEM containing 10% FBS and incubated in a 37°C incubator with 5% CO2. Infection with adeno associate virus (AAV) was performed the following day. AAV vectors were genetically engineered to express either *Gng7* Transcript 1 (AAV2/2CMVGng7), Transcript 3 (AAV2/9CMVGng7var3) or an artificial construct containing only the *Gng7* coding region (AAV2/9CMVGng7). The packaging, purification, and determination of vector titers were performed by the University of Iowa Vector Core (Iowa City, IA). An additional AAV encoding *Gnb2* (AAV2LCMVmGnb2) was obtained from VectorBuilder (Chicago, IL). Viral stocks were thawed on ice and the desired amount of virus was added to growth media to achieve AAV particles 1×10^ 9GC/ml (10,000 MOI x 10^6 cells). After 24 h, media was replaced, and cells were incubated for an additional 24 h with another aliquot of AAVs. The following day, the infected cells were collected and pelleted for qPCR and Western blot analyses.

### MicroRNA bioinformatics

To determine miRNA recognition elements within the *Gng7* mRNA 3’UTR, we used the miRDB database (Liu and Wang, 2019; Chen and Wang, 2020). miRNA expression in the brain was predicted by the TissueAtlas database (Saarland University).

### miRNA transfections

HEK293T cells were grown in DMEM containing 10% FBS and incubated in a 37°C incubator with 5% CO2. The following day cells (1 × 10^6 cells) were electroporated with 5 μg of *Gng7* Transcript1 plasmid and 50–200 nm of various miRNA mimics or inhibitors (Dharmacon, Lafayette, CO) using the Neon Transfection System (Thermo Fisher). Neon Transfection parameters were optimized with voltage (1,100 V), duration (20 ms), and pulse (Bustamante et al., 2002). The Neon Transfection 100 μl kit with buffer R was used for these experiments. The total volume of cell solution and transfected materials added did not exceed 120 μl. The transfected HEK293 cells were transferred into 6-cell plates containing 2 ml of pre-warmed, complete DMEM antibiotic-free cell culture medium with 10% FBS. The cells were grown in the culture for 48 h, harvested, and processed for qPCR.

### Luciferase reporter assays

HEK293T cells were seeded into 6-well plates the day before transfection. The *Gng7* 3′UTR luciferase reporter was obtained by cloning the 3′UTR of *Gng7* Transcript 1 (ENSMUSG00000048240, insert size = 2,997 nt) downstream of the firefly luciferase gene (OriGene, Rockville, MD). Co-transfection of 200 ng of *Gng7* 3’UTR luciferase reporter and 100 ng of miRNA mimics (Dharmacon) was performed through electroporation using the Neon Transfection system, as described above. After 48 h, the cells were collected, and luciferase activity was measured on a Clariostar instrument (BMG Labtech) using the Firefly luciferase assay kit, as described by the manufacturer (Origene). Firefly luciferase activity of cells co-transfected with miRNA mimics+*Gng7* 3’UTR reporter was normalized to luciferase activity of cells transfected with control *Gng7* 3’UTR reporter alone.

### Quantitative polymerase chain reaction (qPCR)

For brain samples, mice (*n* = 5–6/group) were euthanized by rapid decapitation, and brains were rapidly extracted. The striatum was dissected under a dissection microscope using fine forceps, snap-frozen in liquid nitrogen and stored at −80°C. Frozen tissue was homogenized on ice with a motorized pestle (Kimble Chase) in Trizol (Invitrogen). For cultured cells, HEK293 cells were harvested and processed using Trizol (Invitrogen). Total RNA was isolated using the direct-zol RNA miniprep kit (Zymo Research). First-strand cDNA was synthetized from 1 μg of RNA using SuperScript IV first-strand synthesis system (Invitrogen), following the manufacturer’s instructions in a T100 Thermal Cycler (Bio-Rad). mRNA expression levels were determined by qPCR using Green-2-Go qPCR Mastermix (Bio Basic) according to the manufacturer’s protocol on the AriaMx Real-time PCR system (Agilent). Primer pairs for the *Gng*7 gene were designed to target unique sequences within the 5’UTRs of different *Gng*7 transcripts or shared coding regions of all transcripts (Table 1). Melting curve analysis (60–95°C) was performed to verify reaction specificity for each PCR product. The expression of all investigated genes was normalized to the endogenous reference gene glyceraldehyde-3-phosphate dehydrogenase (GAPDH) and calculated according to the relative quantification (ΔΔCt) method. To determine the relative abundance of various *Gng7* transcripts, absolute quantification qPCR was performed. RNA quantification was performed using a ND-1000 spectrophotometer (NanoDrop) and RNA isolation samples between 1.8 and 2.1 A_260_/A_280_ ratio were used. A standard curve was produced by 10 fold serial dilution of a RNA standard sample (1:10, 1:100, 1:1000, 1:10,000). Quantification data was determined by Agilent Aria 1.8 software and quantity determined by the equation: Quantity = 10^(Cq-b)/m^, where b is the y-intercept and m is the slope of the linear regression. The units of quantity are defined by the dilutions used to create the standard curve. Copy number of various *Gng7* transcripts was normalized to *Gng7* coding region.

**Table 1:** Forward and reverse primers for RT-qPCR.

**Mouse gene**	**Alignment sequence**	**Forward (5′→3′)**	**Reverse (5′→3′)**	**bp**
**Gng7 coding region**	NM 010319.4	TCATGGCCGATGATGTCAGGTACT	ATGCGTTCGATCCCAGCTTCAATG	89
**Gng7 transcript 1**	NM_010319.4	TGCTGAGACACTGGCTGGA	ATGCGTTCGATCCCAGCTTCAATG	189
**Gng7 transcript 2**	NM_001038655.2	ATGTCAGCGTGGGAAGGGAA	ATGCGTTCGATCCCAGCTTCAATG	249
**Gng7 transcript 3**	ENSMUSG00000048240	ATCCAGAGACCCAGACACTA	ATGCGTTCGATCCCAGCTTCAATG	235
**Gnal**	NM_010307.3	GTACTATCATACCTCCAGTTCCAC	AGCCCAGGATGCCCTTTAGT	150
**Gapdh (mouse)**	NM_001411844.1	TGCCAAGTATGATGACATCAAGAAG	AGACTCGGGCCATTGTTTCTGTG	71
**GAPDH (human)**	NM 001289745.3	CGCTCTCTGCTCCTCCTGTT	CCATGGTGTCTGAGCGATGT	81

### Membrane preparation

HEK293 cells or striatal tissue were lysed in HME with protease inhibitors (20 mm HEPES, pH 8.0, 2 mm MgCl_2_, 1 mM EDTA, complete EDTA-free protease inhibitor cocktail) and then repeatedly passed through a 25-gauge needle on ice. Nuclei and unbroken cells were pelleted by low-speed centrifugation (250 g) for 5 min, and crude membranes were collected by ultracentrifugation at 250,000 g at 4°C for 30 min, resuspended in 100–200 μl HME with proteinase inhibitors, aliquoted, and then stored at −80°C until use. The protein concentrations of membrane extracts were determined by Pierce 660 nm Protein Assay Reagent (Thermo Fisher Scientific).

### Western blot

Equal amounts of proteins (25 μg/well) were loaded onto 12% Nu-PAGE Bis-Tris gels (Invitrogen, Thermo Fisher Scientific) and transferred to PVDF membranes (Bio-Rad). Total protein staining was determined using REVERT total protein stain (LI-COR Biosciences) and used as a loading control. After 20 min blocking with 10% milk in TBS with 0.01% Tween 20 (TBST), the blots were incubated overnight in 3% milk-TBST with rabbit polyclonal anti- γ_7_ (1:500) (Schwindinger et al., 2003), rabbit polyclonal anti-α_olf_ (1:4000) (Corvol et al., 2001) and mouse monoclonal anti-AC5 (Xie et al., 2015). After three successive washes with TBST, the blots were incubated for 2 h in 3% milk-TBST with HRP-conjugated goat anti-rabbit or anti-mouse secondary antibodies (1:10,000, Jackson ImmunoResearch Laboratories, #111–035-003 and #111–036-003). Western blots were visualized and quantitated by enhanced chemiluminescence (Thermo Fisher Scientific) using an LI-COR Odyssey Fc imager.

### Basescope *in situ* hybridization

Mice (*n* = 3) were killed by rapid decapitation. The brain was readily extracted, snap-frozen in isopentane, chilled with dry ice, and stored at −80°C. Tissues were then frozen in O.C.T. (Sakura Finetek), and 15 μm coronal cryosections of the striatum were collected on Superfrost Plus microscope slides (Thermo Fisher Scientific) and stored at −80°C until use. *In situ* hybridization was performed using the Basescope Duplex Reagent Kit (Advanced Cell Diagnostics, #323800), which allows the detection of multiple target mRNAs at a single cell resolution with appropriate spatial context (Courtney et al., 2018). The Basescope probes were designed to target unique sequences in the non-coding regions of Transcript 1 (Mm-*Gng7* #1058411-C2-Red) or Transcript 3 (Mm-*Gng7* #1059961-C1-Green). Probes were designed and provided by the manufacturer, and the experimental procedure followed the manufacturer’s instructions. Hematoxylin was used for nuclear counterstaining. Brightfield images were acquired using a 20x objective (CFI Plan Apo, Nikon) and Nikon Elements capture software on a Nikon A1R confocal microscope in the FAU Brain Institute Cell Imaging Core. Positive and negative control probes were used to confirm the preservation of sample mRNA and establish nonspecific labeling. Quantitative analysis was performed using ImageJ Fiji (National Institute of Health). A 200 × 200-pixel section with nonoverlapping cells was selected. Positive probe signal was identified as punctate red (Transcript 1) or green (Transcript 3) dots. Dots overlapping with the hematoxylin staining or surrounding the perinuclear space were considered cytosol-localized, while dots outside the hematoxylin staining were considered neuropil-localized. Cytosol and neuropil-localized dots were represented as a percentage of total dots counted for that section. Counts from 4 brain sections/mouse were averaged.

### Golgi staining

Mice (*n* = 3–4 × genotype × sex) were killed by rapid decapitation, the brains were readily extracted, placed in Golgi-Cox solution, and stored in the dark. After 14 days, the brains were transferred in 30% sucrose solution and allowed to sit in the dark for 3 days. Brains were snap-frozen in isopentane chilled with dry ice and stored at −80°C. Hundred μm coronal cryosections of the striatum were collected on gelatin-coated microscope slides (Thermo Fisher Scientific) and stored at −80°C until use. Staining procedure followed the FD Rapid Golgistain Kit (FD Neurotechnologies, Inc.) manufacturer’s instructions. Bright-field images of Golgi-Cox impregnated MSNs and GCs were acquired with a Nikon confocal microscope (60X oil objective) taken in 30–40 μm z-stacks (1 μm step). Dendritic spines were manually counted in the proximal parts of secondary dendrites and normalized to the lengths of the analyzed dendrites. All small protrusions on dendrites were considered as spines. Dendritic spine density was calculated as an average number of spines per 1 μm. To measure spine diameter, a line was manually drawn across the head of each spine, and measurements were obtained using Fiji. Values obtained from each dendritic segment were averaged. Approximately a 50 μm dendritic segment on each neuron was analyzed and a total of 4–5 neurons per mouse were examined. Each data point represents spine density or spine diameter per neuron.

### Cocaine locomotor sensitization

Behavioral testing was performed in the Neurobehavior Core facility of the Florida Atlantic University Brain Institute. Animals were handled and habituated to the injection procedure by saline intraperitoneal (i.p.) administration for 3 days preceding the experiment. Mice were habituated for 2 h to the testing room the day before testing and at least 30 min before the start of the experimental procedures. Male and female mice were tested on separate days by an experimenter blinded to animal genotypes. Locomotor activity was detected using a photocell-based automated monitoring system (Med Associates, Inc) and measured as distance traveled (cm) in 5 min intervals. Vertical activity was detected by a third array at 4 cm above the arena floor and measured as the number of rearing in 5 min intervals. Med Associates Open Field Activity software was used to track and analyze mouse movements. Cocaine sensitization was induced using the two-injection protocol as described by Valjent et al. (2009). Mice were habituated to the test apparatus for 2 h on day 1. On day 2, spontaneous activity was recorded for 30 min, then mice received an injection of saline, and their activity was recorded for an additional 90 min. On day 3, mice underwent the same protocol as day 2, but they were administered 20 mg/kg cocaine (Sigma). Seven days later (day 10), mice received the second dose of cocaine. Cumulative distance traveled during the 90 min after injection on both days 3 and 10 was considered for data analysis of cocaine-induced locomotor sensitization. Cocaine solution and saline were injected i.p. in a volume of 5 ml/kg.

### Statistical analysis

All statistical analyses were performed using GraphPad Prism 8 (GraphPad Software). Data are expressed as mean ± SEM. n refers to the number of independent measures. *p-*values ≤0.05 were considered statistically significant. All animal studies involved balanced groups of male and female mice, and data from each sex were analyzed separately. Parametric statistics were used based on standard data distribution, as confirmed by the Shapiro–Wilk test.

## Results

### *Gng7* gene structure

**Figure 1:**
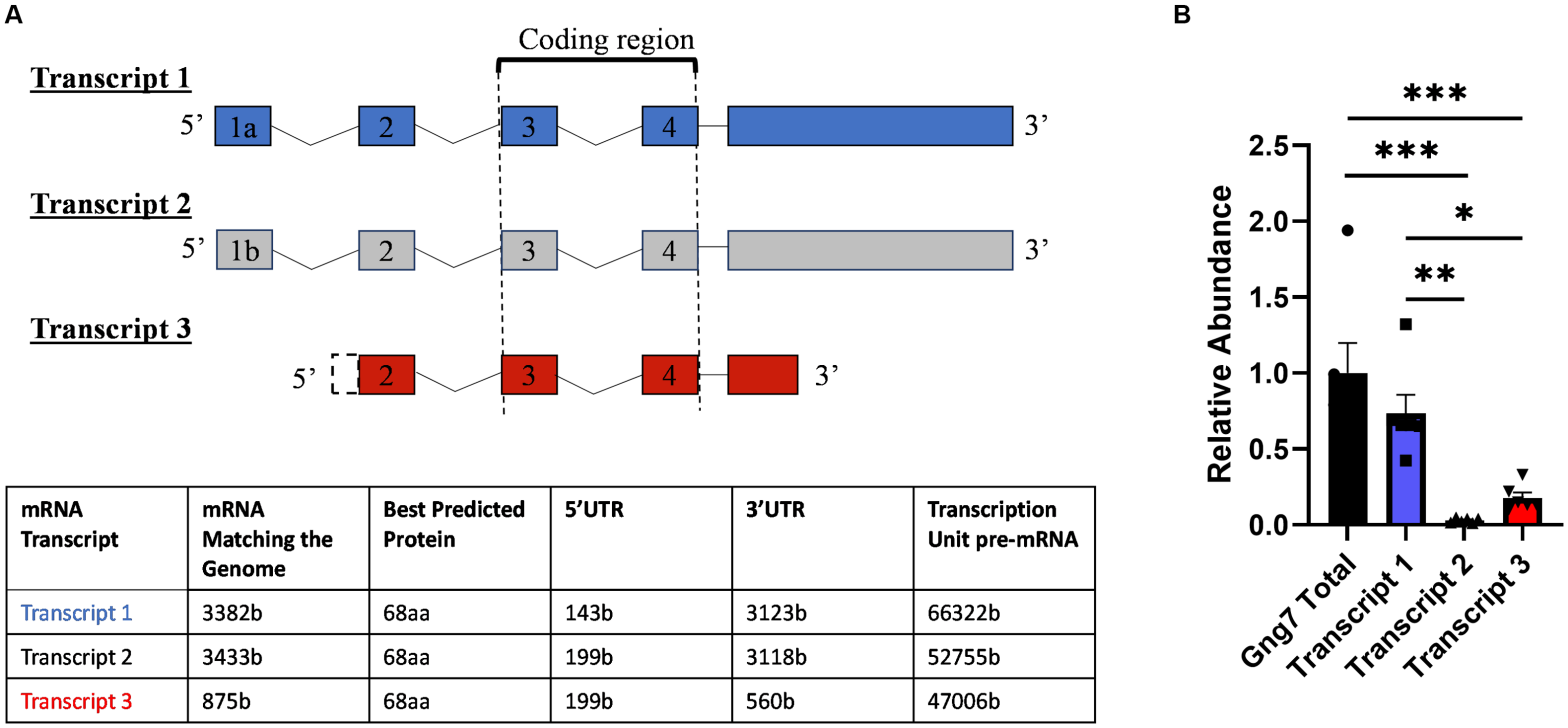
Mouse Gng7 gene splicing variants and their relative expression in the striatum. **(A)** Transcripts 1 and 2 arise from using alternative exons 1a and 1b, respectively, and both share a 3 kb 3’UTR. Transcript 3 arises from exon 2 and is characterized by shorter 560-bp 3’UTR. Exons 3–4 represent coding region, which is conserved among transcripts. **(B)** qRT-PCR revealed Transcript 1 was the predominant species, accounting for >90% of the total *Gng7* level in the striatum, followed by transcript 3 and transcript 2 (one-way ANOVA; *p < 0.05, **p < 0.01,***p < 0.001). *n* = 6 mice.

The mouse *Gng7* gene is surprisingly large and complex. Spanning >66 kb on mouse chromosome 10, this gene utilizes alternative promoters and polyadenylation sites to produce three major mRNA transcripts encoding same 68-amino acid protein (Figure 1A). Transcripts 1 (ENSMUST00000117805.8) and 2 (ENSMUST00000118233.8) arise from the usage of alternative exons 1a (90b) and 1b (146b), have the same exon 2 (53b) and display a long 3’UTR (>3-kb). Transcript 3 (ENSMUST00000118465.8) arises from a promoter region upstream of exon 2, has an increased length exon 2 (199b), and has a short 3’UTR (560b).

Using real-time reverse transcription (RT), quantitative PCR (qPCR), we determined the relative abundance of the three mRNA transcripts in the adult striatum. For this purpose, primer pairs were targeted against unique sequences within the 5’UTRs of the individual transcripts along with another set of primers targeted to the shared coding region of all transcripts. This analysis revealed that Transcript 1 was the most abundant species in adult striatum, accounting for >90% of the total *Gng7* level (Figure 1B). The remainder was comprised of Transcript 3 with barely detectable levels of Transcript 2. The qPCR products amplified by each primer pair were confirmed to share the *Gng7* coding region by sequencing.

**Figure 2:**
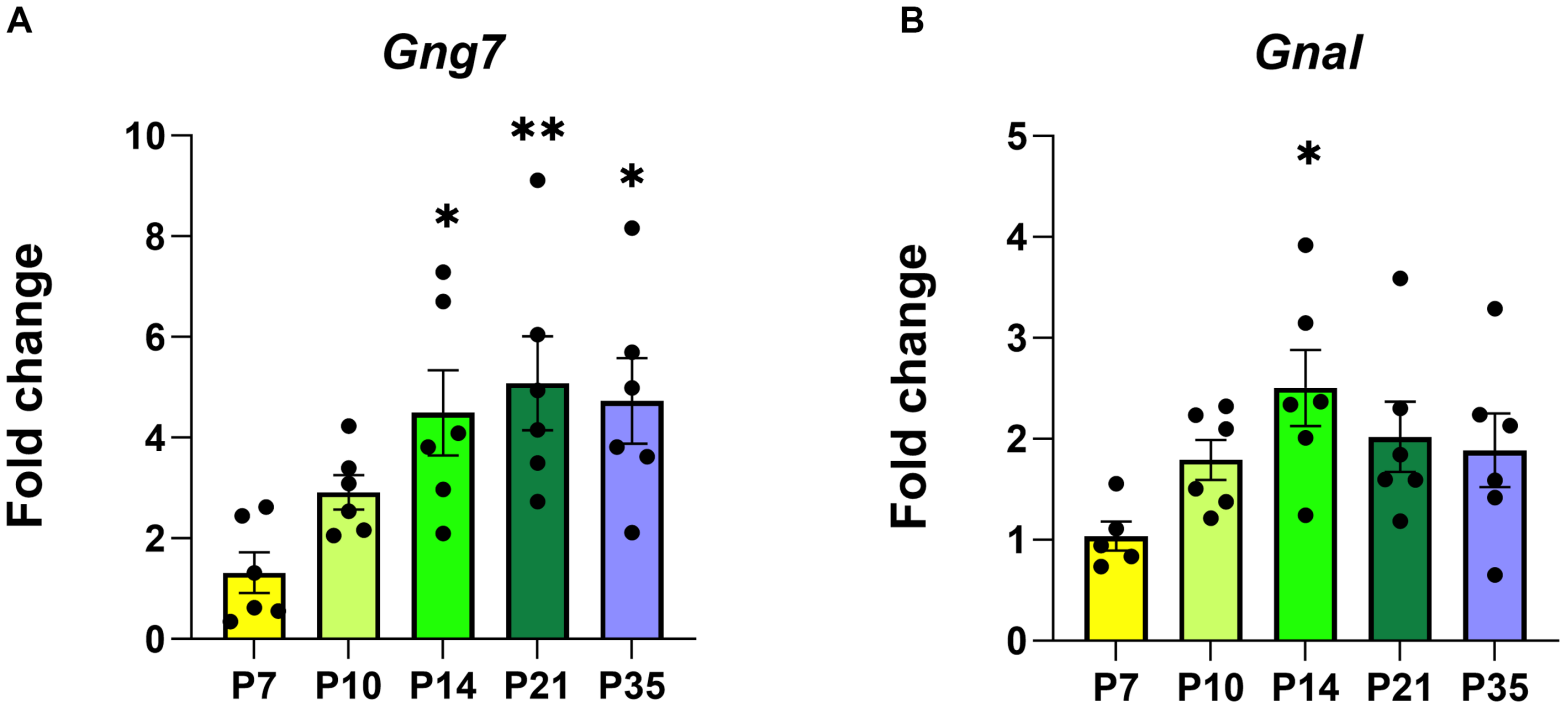
The postnatal developmental *Gng7* and *Gnal* mRNA levels in *Gng7^+/+^* mouse striatum. **(A)** *Gng7* and **(B)** *Gnal* expression in the striatum at five developmental time points (P, postnatal day). Values are expressed as mean ± SEM. Asterisks indicate a statistically significant difference from the P7 time point (one-way ANOVA; *p < 0.05, **p < 0.01). *n* = 5–6 mice/time point.

In humans and mice (Fentress et al., 1981; Jain et al., 2001), the striatum forms during the immediate post-natal period (Fishell and Van Der Kooy, 1991; Lieberman et al., 2018). During this timeframe, the expression of D_1_R, D_2_R, and A_2a_R receptors increases dramatically coinciding with key aspects of striatal functions (Novak et al., 2013; Lebouc et al., 2020). In this study, we investigated the temporal expression patterns of both the *Gng7* and *Gnal* (Rius et al., 1994; Xie and Martemyanov, 2011) genes encoding the γ_7_ and α_olf_ proteins during five critical developmental windows (Figure 2A and 2B). Using similar RT-qPCR analyses, both gene transcripts are detected as early as postnatal day 7 (P7), at a time when mouse pups show little movement, striatal neurons are just forming, and glutamatergic inputs are minimal. Their expression levels continue to rise through postnatal days 10 to 14 (P10, P14), at a stage when mouse pups exhibit increased motor behaviors and glutamatergic and dopaminergic inputs are undergoing rapid maturation. Finally, their expression levels plateau between postnatal days 21 to 35 (P21, P35), at a time when mice are displaying the full range of locomotor and learned behaviors and the balance among various inputs has achieved maturity (Krajeski et al., 2019). *Gng7* transcript 1 expression follows a similar pattern as the *Gng7* shared coding region, suggesting that transcript 1 is the prevalent variant throughout each developmental state (Supplementary Figure S1). Thus, the *Gng7* and *Gnal* genes appear to be co-transcriptionally regulated along with other components of their associated signaling pathways and functional outputs (Schwindinger et al., 2012; Novak et al., 2013), during striatal development.

Subsequently, Western blot analysis of striatal membranes showed that protein levels of α_olf_, γ_7,_ and AC5 (Supplementary Figure S2) follow a similar temporal expression pattern (Rius et al., 1994; Xie and Martemyanov, 2011). Altogether, the synchronized expression of these genes and their associated proteins underscores the assembly of a specific Gα_olf_β_2_γ_7_ signaling complex and focuses attention on the intricate processes required for its specific assembly and functioning during striatal development.

### Post-transcriptional regulation of *Gng7* expression

Our findings above reveal that *Gng7* expression is tightly regulated with respect to both striatal development and function. As the predominant species in this brain region, Transcript 1 is notable for its use of an alternative transcription initiation site and the addition of a very long 3′ untranslated region (3’UTR). This latter feature is characteristic of certain brain mRNA transcripts that utilize extended 3′UTRs to impart post-transcriptional regulation of protein abundance and/or subcellular localization (Wein et al., 2003; An et al., 2008; Malka et al., 2017; Tushev et al., 2018; Mayr, 2019; Bae and Miura, 2020; Cheng et al., 2021).

**Figure 3:**
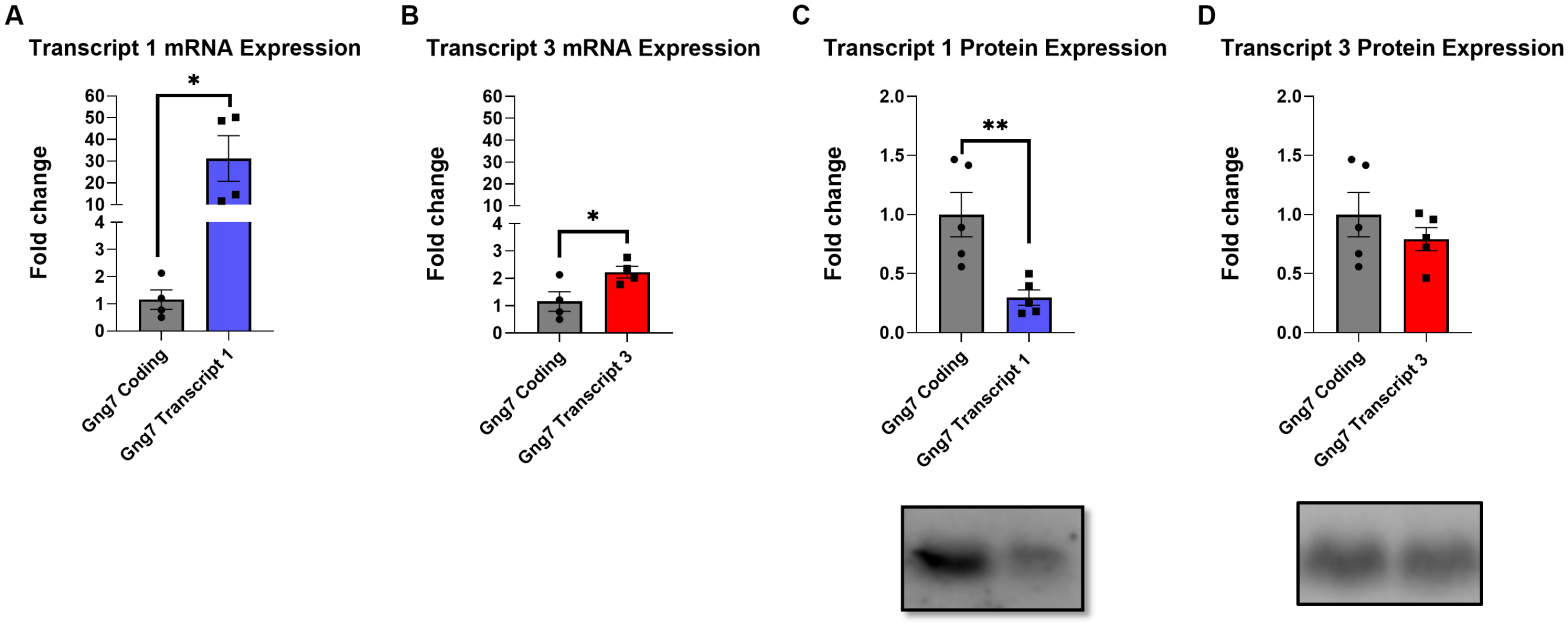
*In-vitro* infection of AAVs encoding *Gng7* transcripts in HEK293T cells. **(A)** Gene expression of *Gng7* after infection with AAVs encoding *Gng7* coding region and *Gng7* transcript 1 or **(B)** *Gng7* transcript 3. **(C)** γ_7_ protein expression after infection with *Gnb2* AAV plus AAVs encoding *Gng7* coding region and *Gng7* transcript 1 or **(D)** *Gng7* transcript 3 AAV. Values are normalized to gene or protein expression in cells infected with AAV encoding *Gng7* coding region and shown as fold changes. Values are expressed as mean ± SEM. Asterisks indicate a statistically significant difference from *Gng7* coding (student’s *t*-test; *p < 0.5, **p < 0.1). *n* = 4–5 well/group.

To investigate this possibility, we used two complementary approaches. For the initial studies, we determined *Gng7* expression levels in HEK293T cells infected with adeno-associated viral (AAV) vectors expressing either Transcript 1, Transcript 3, or an artificial construct containing only the coding region. Quantitative PCR analysis showed significantly higher mRNA levels of Transcript 1 (>20fold), compared to either Transcript 3 or the coding region construct (Figures 3A,B). Subsequently, HEK293T cells were co-infected with an AAV construct expressing the *Gnb2* transcript to produce a stable β_2_γ_7_ dimer and prevent the degradation of the γ_7_ protein (Robishaw and Berlot, 2004; Yim et al., 2019) To assess expression of γ_7_ protein, Western blot analysis was used revealing, despite its higher mRNA level, the Transcript 1 construct produced a significantly lower amount of γ_7_ protein (~70%) than either the Transcript 3 or coding region construct (Figures 3C,D). These findings suggest that Transcript 1 may contain regulatory element(s) within its non-coding region(s) that suppress protein translation.

### Identification of potential regulatory elements in the 3’UTR

**Figure 4:**
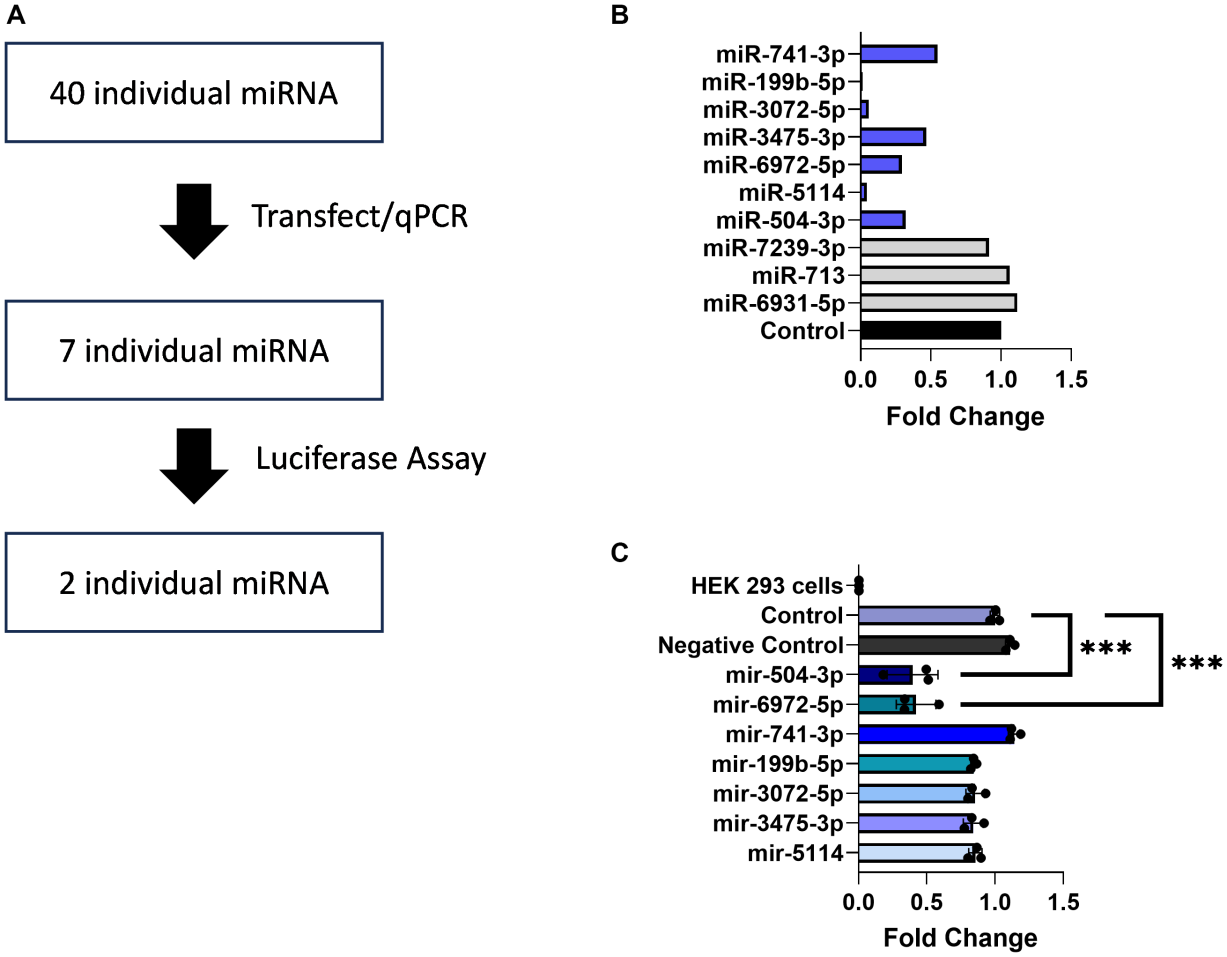
miRNAs binding to the *Gng7* 3’UTR. **(A)** Pipeline of miRNAs screening. **(B)** Gene expression of *Gng7* following co-transfection of *Gng7* vector with selected miRNAs in HEK 293 cells assessed by qPCR. **(C)** Validation of miRNA binding on the *Gng7* 3′UTR by luciferase reporter assay. The mir-504-3p and mir-6972-5p bind to *Gng7* 3’UTR. Results are shown as fold change luciferase signal compared to control (luciferase reporter alone). Values are expressed as mean ± SEM. Asterisks indicate significant difference between control and miRNA mimics (one-way ANOVA; ***p < 0.001). *n* = 3 well/group.

Multiple studies have documented regulatory elements within 3’ UTRs responsible for downregulating gene expression (Mayford et al., 1996; Mori et al., 2000; Blichenberg et al., 2001; Tushev et al., 2018). Prominent among these, miRNAs are small non-coding RNAs of 19–25 nucleotides that interfere with gene expression by binding to the 3′ UTRs of their target mRNAs and acting at the level of translational repression or degradation. The brain expresses the largest number of miRNAs and leverages this regulatory mechanism to fine tune the expression of genes required for neuronal adaptations to changing environmental cues (Bak et al., 2008; Jovičić et al., 2013; Nowakowski et al., 2018). Informatic analysis using the miRDB database identified 102 predicted miRNA binding sites within the 3’UTR of Transcript 1 (Figure 4A). Given this large number, a screening assay was deployed to search for miRNAs capable of downregulating its expression. For this purpose, the TissueAtlas database was used to select 40 of the top target-scoring miRNAs that show expression in the brain and each of the 40 miRNA mimics was tested separately for its ability to impact expression (Supplementary Figure S3). From this screen, seven miRNA mimics, including mir-504-3p, mir-5114, mir-6972-5p, mir-3475-3p, mir-3072-5p, mir-199b-5p, and mir-741-3p, were identified that resulted in a >50% reduction in Transcript 1 expression (Figure 4B).

**Figure 5:**
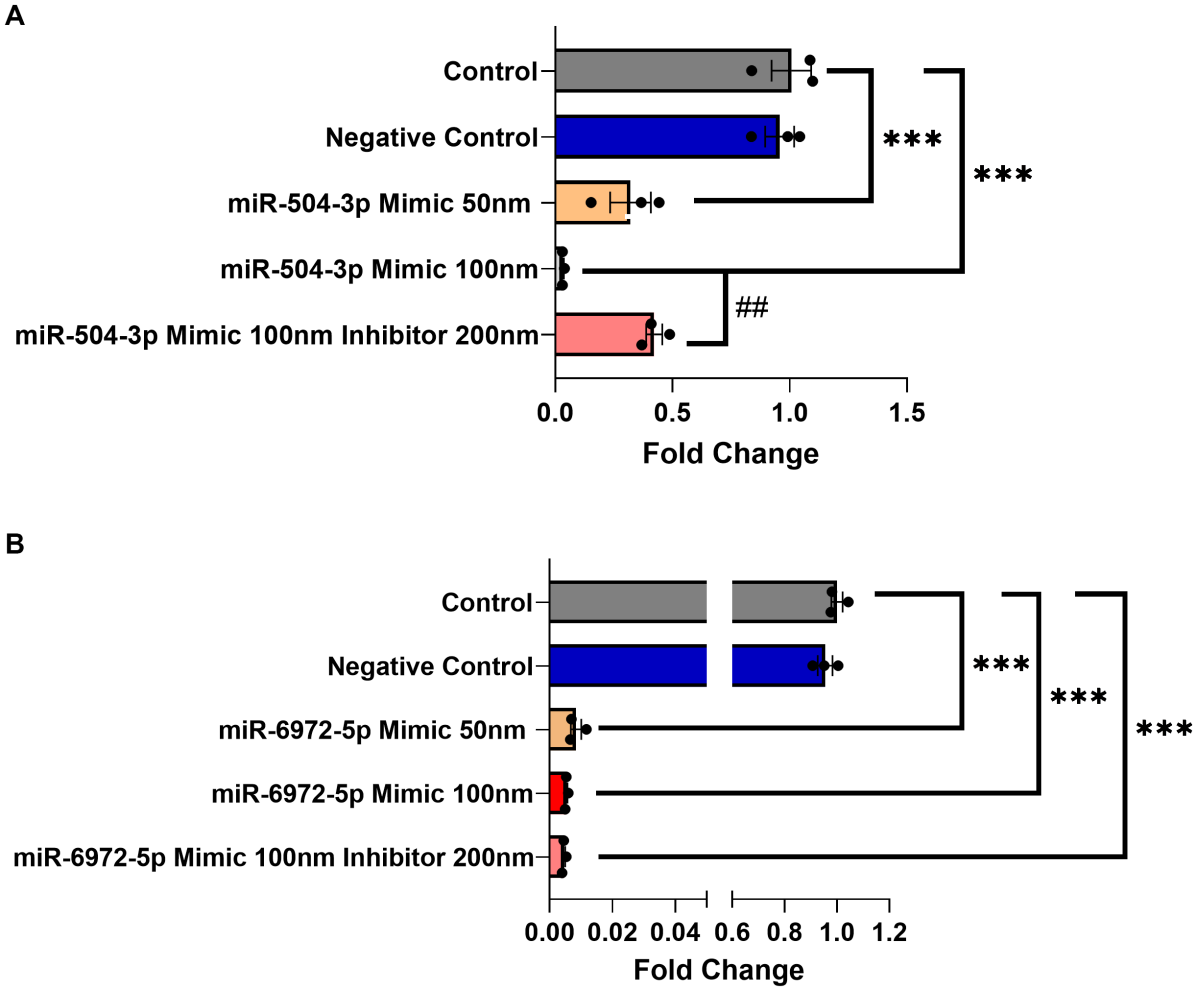
Dose-dependent *Gng7* expression modulation by mir-504-3p and mir-6972-5p in HEK293 cells. **(A)** The miR-504-3p mimic significantly downregulated expression of *Gng7* in a dose-dependent fashion. The miR-504-3p inhibitor partially rescued *Gng7* expression. **(B)** The miR-6972-5p mimic show repression of *Gng7* expression at all doses. Values are expressed as mean ± SEM. Asterisks indicate a statistically significant difference between control and miRNA mimics (one-way ANOVA;***p < 0.001). Pound indicates a statistically significant difference from inhibitor and mimic combination (one-way ANOVA; ##*p* < 0.01). *n* = 3 well/group.

Next, a luciferase gene reporter assay was utilized to confirm whether the candidate miRNAs can specifically bind to the 3’UTR of *Gng7* Transcript 1 and regulate its expression. Of the seven miRNAs, only mir-504-3p and mir-6972-5p produced a significant reduction in luciferase activity across a range of doses spanning 50–100 nM, compared to the miRNA vector (Figure 4C). Altogether, the collective findings indicate that both mir-6972-5p and miR-504-3p may be involved in post-transcriptional regulation by binding to the extended 3’UTR of Transcript 1. However, only the miR-504-3p inhibitor led to partial rescue of *Gng7* expression (Figure 5A), while the mir-6972-5p inhibitor did not at the tested doses (Figure 5B).

### Subcellular localization of γ7 transcripts

**Figure 6:**
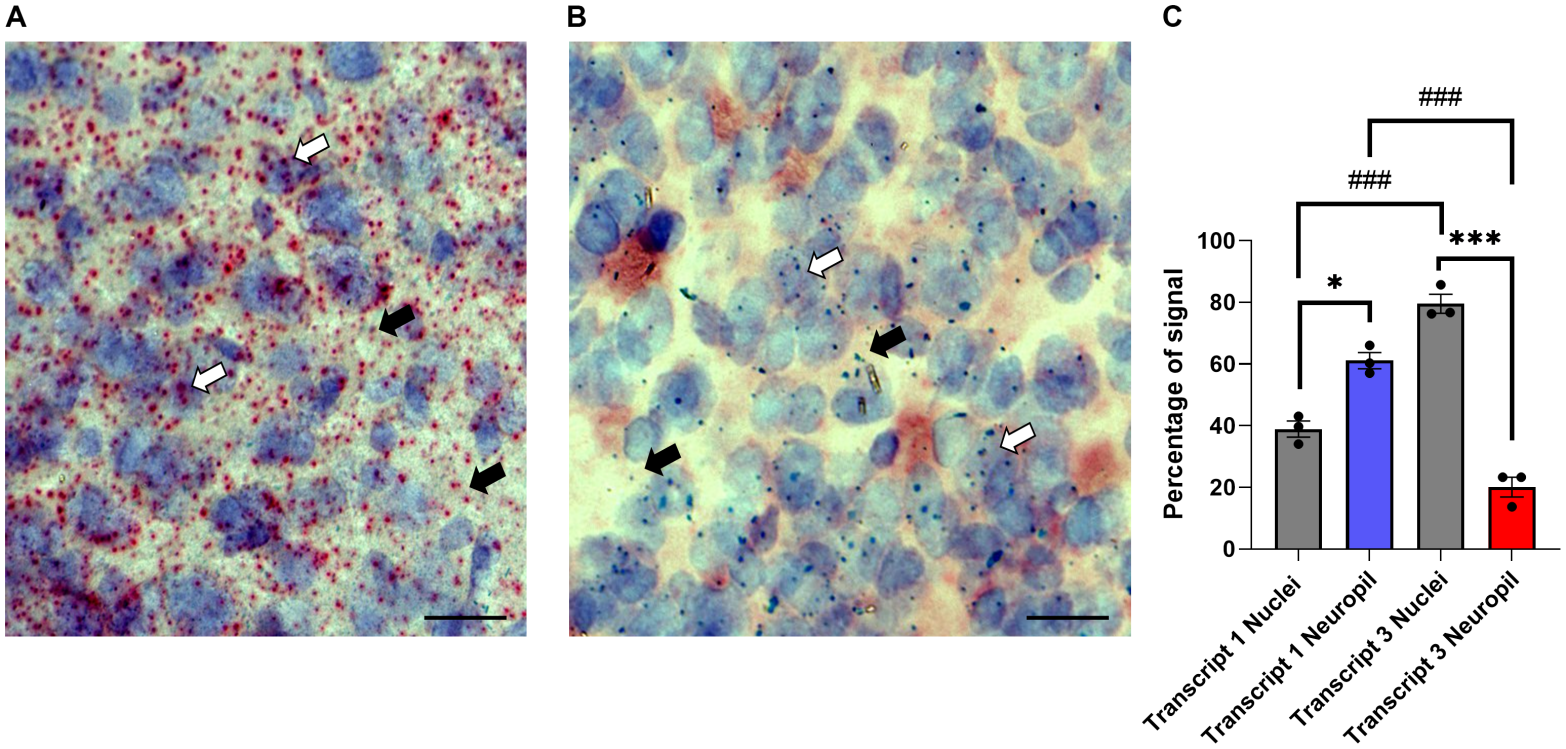
Subcellular localization of *Gng7* transcripts. Basescope® *in situ* hybridization probing *Gng7* transcripts 1 (red label, **A**) and 3 (green label, **B**) in the striatum. Hematoxylin staining (purple label) was used to label cell nuclei. Representative images of 20X magnification widefield (scale bar = 25 μm). White arrow probe co-localized with hematoxylin staining, black arrow probe in the neuropil. **(C)** Quantification of transcript 1 and 3 localization in nucleus vs. neuropil. Values are expressed as mean ± SEM. (Repeated measures two-way ANOVA and Bonferroni *post-hoc*; *p < 0.05, ***p < 0.001 neuropil vs. nuclei ###p < 0.001 Transcript 1 vs. Transcript 3). *n* = 3 mice/group.

Translational repression is often seen for neuronal transcripts that are transported to dendrites (Martin and Zukin, 2006; Wells, 2006; Rodriguez et al., 2008; Fonkeu et al., 2019). While previous studies showed the γ_7_ mRNA(s) is highly localized to dendrites (Watson et al., 1994; Tushev et al., 2018), information on the individual transcripts and their subcellular distribution is lacking. For this purpose, coronal cryosections of the dorsal and ventral striatum from wildtype *Gng7*^+/+^ mice were prepared for *in situ* hybridization using the Basescope technology. The Basescope probes were designed to target unique sequences in the non-coding regions of Transcript 1 or Transcript 3. Qualitative assessment of Transcript 1 distribution revealed abundant signal throughout the entire striatum, consistent with the high copy number of this transcript (Figure 6A). Transcript 1 showed an evenly dispersed distribution in areas coinciding with hematoxylin staining as well as in the surrounding regions, likely corresponding to the neuropil compartment (Figure 6A). In contrast, sections probed for Transcript 3 revealed a sparse signal overlapping or near the hematoxylin nuclear staining, likely corresponding to its cytoplasmatic localization (Figure 6B). The quantitative analyses confirmed Transcript 1 copies were significantly enriched in the neuropil compared to cell nuclei, whereas Transcript 3 copies were localized near cell soma (Figure 6C). The findings suggest that Transcript 1 plays a critical role in modulating the γ_7_ protein level and driving the stoichiometric assembly and function of the G_olf_ heterotrimer in MSN dendrites.

### *Gng7^−/−^* mice have a change in dendritic morphology

In the first part of our study, we demonstrate γ_7_ protein production and hence G_olf_ assembly are primarily concentrated in the neuropil, and more specifically, within dendritic spines of medium spiny neurons. By transmitting signals from D_1_ dopamine (D_1_R) and A_2a_ adenosine (A_2a_R) receptors to their downstream effectors, this unique population of postsynaptic G_olf_ protein may be strategically positioned to regulate the excitability and synaptic characteristics within the direct and indirect pathways. Although the molecular mechanisms governing the production and signaling of the G_olf_ protein within the spines have been a mystery, our findings highlight the significance of a predominant *Gng7* splice variant with an unusually extended 3’UTR that is targeted by specific miRNAs to regulate localized γ_7_ protein production. This initial discovery identifies a possible miRNA-mediated process to dynamically regulate the amount of the functional G_olf_ protein within the spines of medium spiny neurons where the majority of glutamatergic synapses are formed. Because the G_olf_ protein represents the rate-limiting step for these two downstream signaling pathways, we hypothesize that this regulatory mechanism may impact synaptic morphology and/or function implicated in drug addiction. Accordingly, we used both global and conditional knockout mice to address this hypothesis.

**Figure 7:**
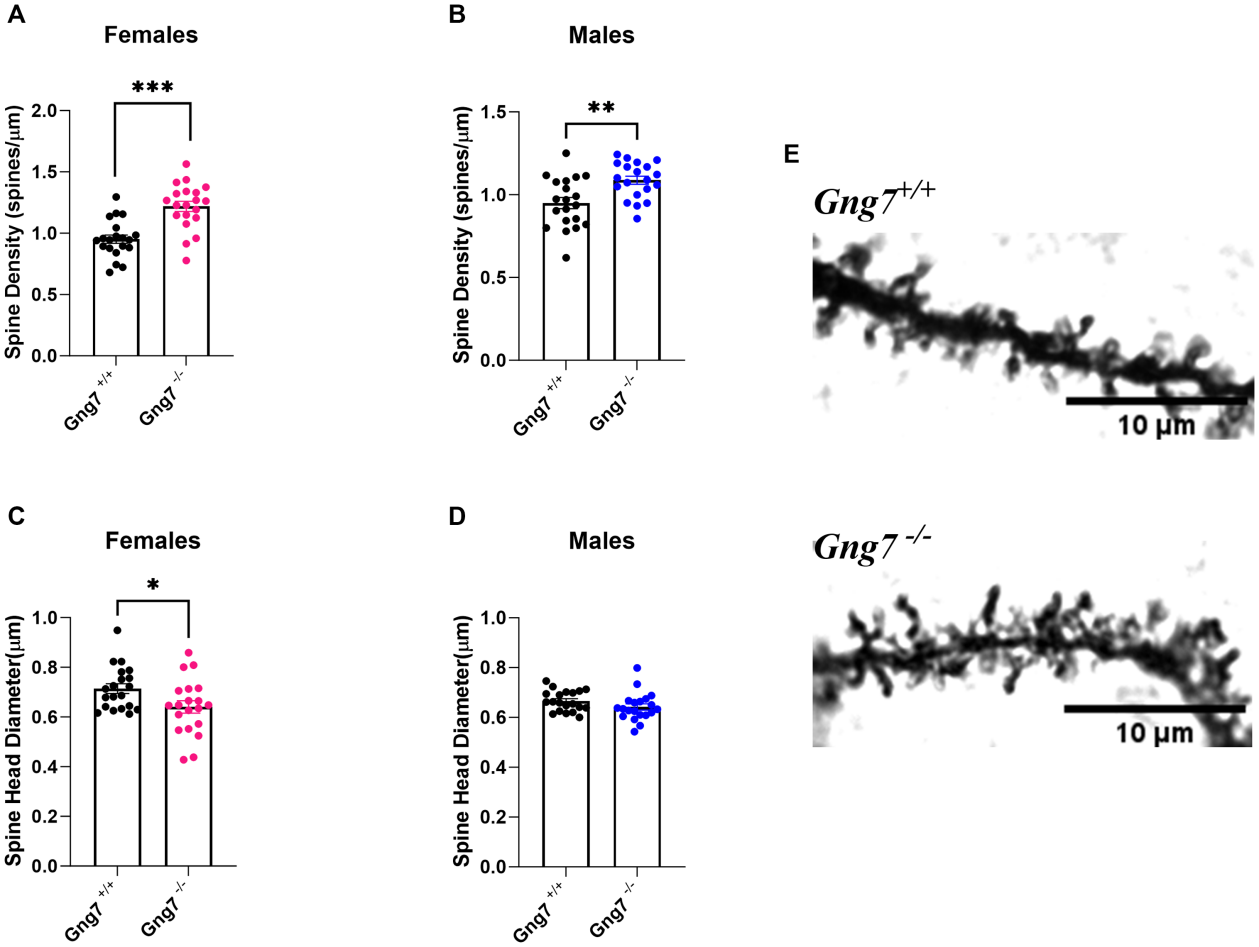
Dendritic morphology of striatal medium spiny neurons in *Gng7^−/−^ and* Gng7^+/+^ wildtype mice. **(A,B)** Analysis of MSN dendrites in both male and female *Gng7^−/−^* mice revealed a significant increase in dendritic spine density compared to *Gng7^+/+^* wildtype mice. **(C)** MSNs of female *Gng7^−/−^* mice displayed significant decrease in spine head diameter. **(D)** MSNs of male *Gng7^−/−^* mice did not show significant change in spine head diameter. **(E)** Representative MSN dendritic segments from *Gng7^+/+^* and *Gng7^−/−^* mice used for analysis of spines. Values are expressed as mean ± SEM. Asterisks indicate a statistically significant difference from *Gng7^+/+^* (student’s *t*-test; *p < 0.05, **p < 0.01 ***p < 0.001). *n* = 20 neurons/group.

Comprising more than 90% of striatal neurons, the MSNs are distinguished by their dense dendritic arbors and spines that represent the morphological sites for incoming glutamatergic and dopaminergic signals (Zhou, 2020). MSN dendritic spines undergo intense remodeling during early stages of postnatal development, with initial overgrowth followed by pruning and maturation in adulthood. Notably, changes in dendritic spine formation and associated alterations in circuit output have been linked to a variety of neuropsychiatric disorders including addiction (Johnston et al., 2016; Forrest et al., 2018; Neha, 2020). To determine how gene-targeted loss of *Gng7* affects dendritic growth and distribution of dendritic spines, morphological analyses of Golgi-stained MSNs were performed on brain sections from *Gng7*^−/−^ knockout and wildtype mice. We found MSNs from both male and female *Gng7*^−/−^ mice exhibited a significantly higher density of dendritic spines (Figures 7A,B). In addition, MSNs from *Gng7*^−/−^ female mice showed a significant reduction in spine head diameter (Figures 7C,D). Altogether, these findings suggest that the elimination of the γ_7_ protein and the resulting loss of the G_olf_ signaling complex (Schwindinger et al., 2003, 2010) are important for spine production and/or pruning in MSNs.

### Cocaine sensitization in global and conditional Gng7 knockout mice

Repeated exposure to drugs of abuse results in augmented motor stimulant effects elicited by subsequent drug exposure (Vanderschuren and Kalivas, 2000) and is associated with long-lasting alterations in intracellular signaling pathways and dendritic morphology of MSNs (Lee et al., 2006; Allichon et al., 2021). Considering the role of Gα_olf_β_2_γ_7_ heterotrimer in dopaminergic signaling and its involvement in shaping MSNs dendritic morphology, we investigated the effects of global *Gng7* deletion on behavioral sensitization to cocaine. *Gng7^+/+^* and *Gng7*^−/−^ mice showed a similar increase in locomotor activity as a result of cocaine administration compared to saline (Supplementary Figure S4) Following a second cocaine injection administered 1 week apart, both wildtype and *Gng7*^−/−^ mice of both sexes showed significant locomotor sensitization [repeated measures two-way ANOVA- MALES: sensitization effect: *F*(1,26) = 31. 6, *p* < 0.001; genotype effect: *F*(1,26) = 0.45, ns; interaction: *F*(1,26) = 1.61, ns; FEMALES: sensitization effect: *F*(1,14) = 138.4, *p* < 0.001; genotype effect: *F*(1,14) = 0.02, ns; interaction: *F*(1,14) = 0.13, ns] (Supplementary Figure S7). Likewise, the distance traveled during the acclimation session and after saline injection were similar between genotypes.

**Figure 8:**
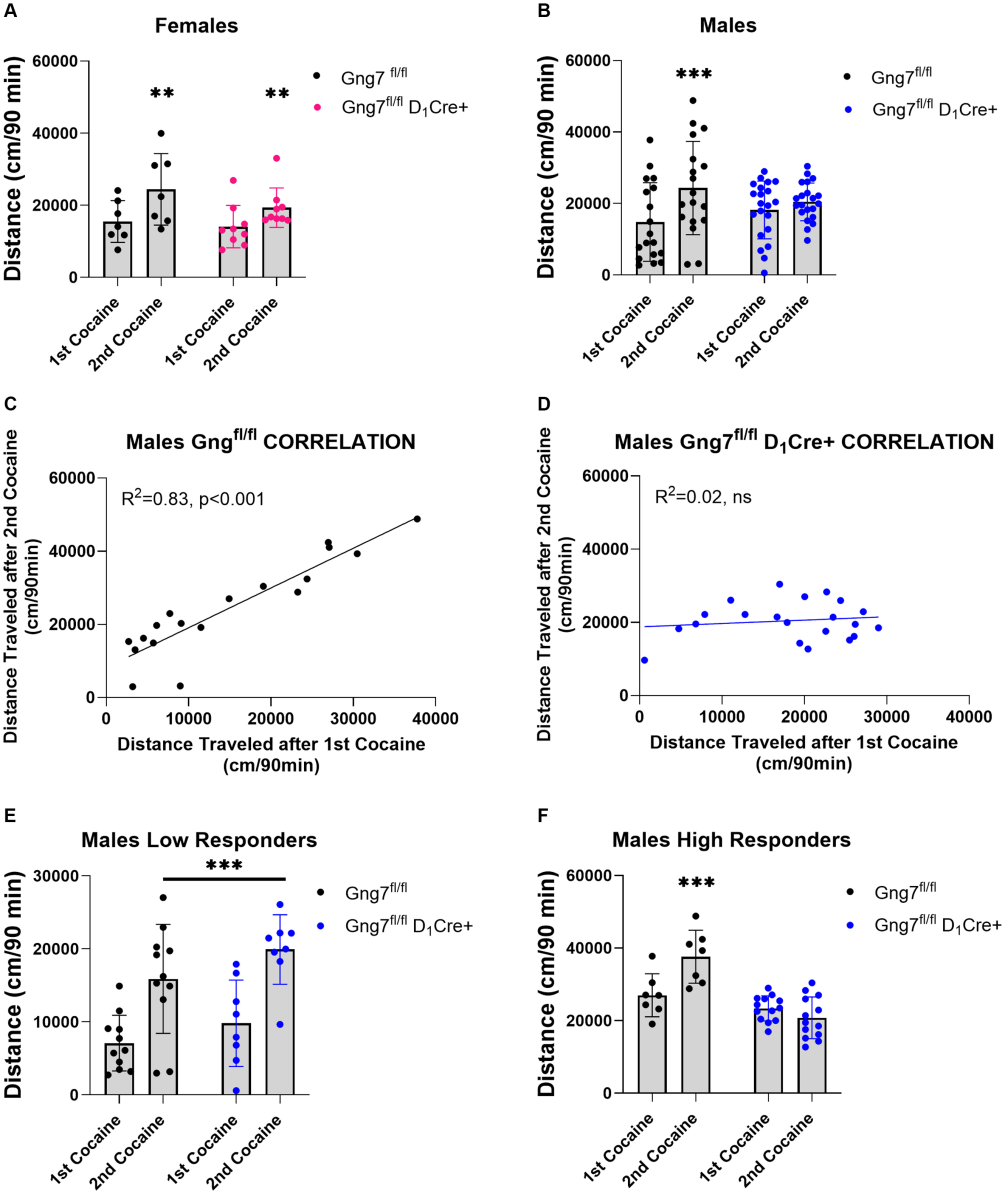
Locomotor sensitization induced by cocaine in *Gng7^fl/fl^* D_1_Cre *+* and *Gng7^fl/fl^* mice. Open field locomotor activity of *Gng7^fl/fl^* and *Gng7^fl/fl^* D_1_Cre + mice following two cocaine injections. Locomotor responses were measured during 90 min after two cocaine injections administered 1 week apart. **(A)** Gng7^fl/fl^ and Gng7^fl/fl^ D_1_Cre + female mice showed significant locomotor sensitization following the second cocaine injection. **(B)** Male Gng7^fl/fl^ mice showed significant sensitization during the second cocaine exposure, however, no sensitization was observed in male Gng7^fl/fl^ D_1_Cre+. **(C,D)** Correlation analysis depicting the acute (*x* axis) and sensitized locomotor (*y* axis) responses to cocaine for Gng7^fl/fl^ and Gng7^fl/fl^ D_1_Cre + male mice. A simple linear regression was fitted to the data to determine the goodness of fit and the degree to which the slope significantly deviates from 0 (*R*^2^). Male Gng7^fl/fl^ mice showed a positive correlation between the responses during the first and second cocaine administration, indicative of cocaine sensitization, while male Gng7^fl/fl^ D_1_Cre + mice did not show no correlation between the two measures. **(E)** Gng7^fl/fl^ and low responder Gng7^fl/fl^ D_1_Cre + male (<18,000 cm during first cocaine) mice displayed significant cocaine locomotor sensitization. **(F)** No sensitization was observed in high responder Gng7^fl/fl^ male mice (>18,000 cm during first cocaine) during the second cocaine injection. Values are expressed as mean ± SEM. (**A,B,E,F** Repeated measures two-way ANOVA, followed, where appropriate, by Tukey’s post-hoc test; **p < 0.01, ***p < 0.001 difference from first cocaine injection). *n* = 7–21 mice/group.

A potential limitation of these studies lies in the global deletion of the γ_7_ protein, which disrupts both D_1_R and A_2a_R signaling pathways that often exert opposing actions. This global effect may have obscured specific findings. To address this, we conducted experiments using conditional knockout mice targeting the *Gng7* gene in distinct striatal neuron subpopulations (Brunori et al., 2021). In initial studies, D_1_R -conditional knockout mice were used to examine the specific contribution of the Gα_olf_β_2_γ_7_ heterotrimer in this pathway on behavioral sensitization to cocaine. Female *Gng7*^fl/fl^ D_1_Cre + and *Gng7*^fl/fl^ control littermates displayed similar acute and sensitized locomotor responses to cocaine repeated measures two-way ANOVA- FEMALES: sensitization effect: [*F*(1,14) = 16.99, *p* = 0.001; genotype effect: *F*(1,14) = 1,12, ns; interaction: *F*(1,14) = 1.14, ns] (Figure 8A and Supplementary Figure S5). Conversely, male mice showed a significant interaction between genotype and sensitization [repeated measures two-way ANOVA- MALES: sensitization effect: *F*(1,37) = 23.5 *p* < 0.001; genotype effect: *F*(1,37) = 0.01, ns; interaction: *F*(1,37) = 8.97, *p < 0.01*]. Tukey’s *post hoc* analysis revealed that male *Gng7*^fl/fl^ control mice exhibited sensitized responses after the second cocaine injection (*p* < 0.001), whereas *Gng7*^fl/fl^ D_1_Cre + did not (Figure 8B). Further analysis confirmed male *Gng7*^fl/fl^ control mice showed a positive correlation between acute and sensitized cocaine responses (*R*^2^ = 0.83, *p* < 0.001, Figure 8C), which was absent in *Gng7*^fl/fl^ D_1_Cre + conditional knockout mice (*R*^2^ = 0.02, ns, Figure 8D).

To further investigate this effect, male mice were divided into two populations based on their overall locomotor activity during the first cocaine session (median = 17,902). Specifically, mice with an initial cocaine response <18,000 cm were classified as low responders, while those with an initial response >18,000 cm were classified as high responders. Separate analyses of cocaine sensitization were performed for each group. In the low responder group, both male *Gng7*^fl/fl^ control and *Gng7*^fl/fl^ D_1_Cre + conditional knockout mice showed significantly sensitized responses to cocaine. The repeated measures two-way ANOVA revealed a significant sensitization effect [*F*(1,17) = 50.7, *p* < 0.001], but no significant genotype effect or interaction (Figure 8E). Conversely, in the high responder group, Tukey’s post-hoc analyses confirmed that male *Gng7*^fl/fl^ D_1_Cre + mice selectively fail to develop a sensitized response to cocaine. The repeated measures two-way ANOVA showed a significant sensitization effect [*F*(1,18) = 0.2, *p* < 0.05] and a significant genotype effect [*F*(1,18) = 24.4, *p* < 0.001], along with a strong interaction [*F*(1,18) = 19.4, *p* < 0.001, Figure 8F]. Notably, the distance traveled during the acclimation session and after saline or first cocaine injection was similar between genotypes (Supplementary Figure S5). These findings identify an essential role for D_1_R signaling through the Gα_olf_β_2_γ_7_ heterotrimer in mediating behavioral sensitization to cocaine.

**Figure 9:**
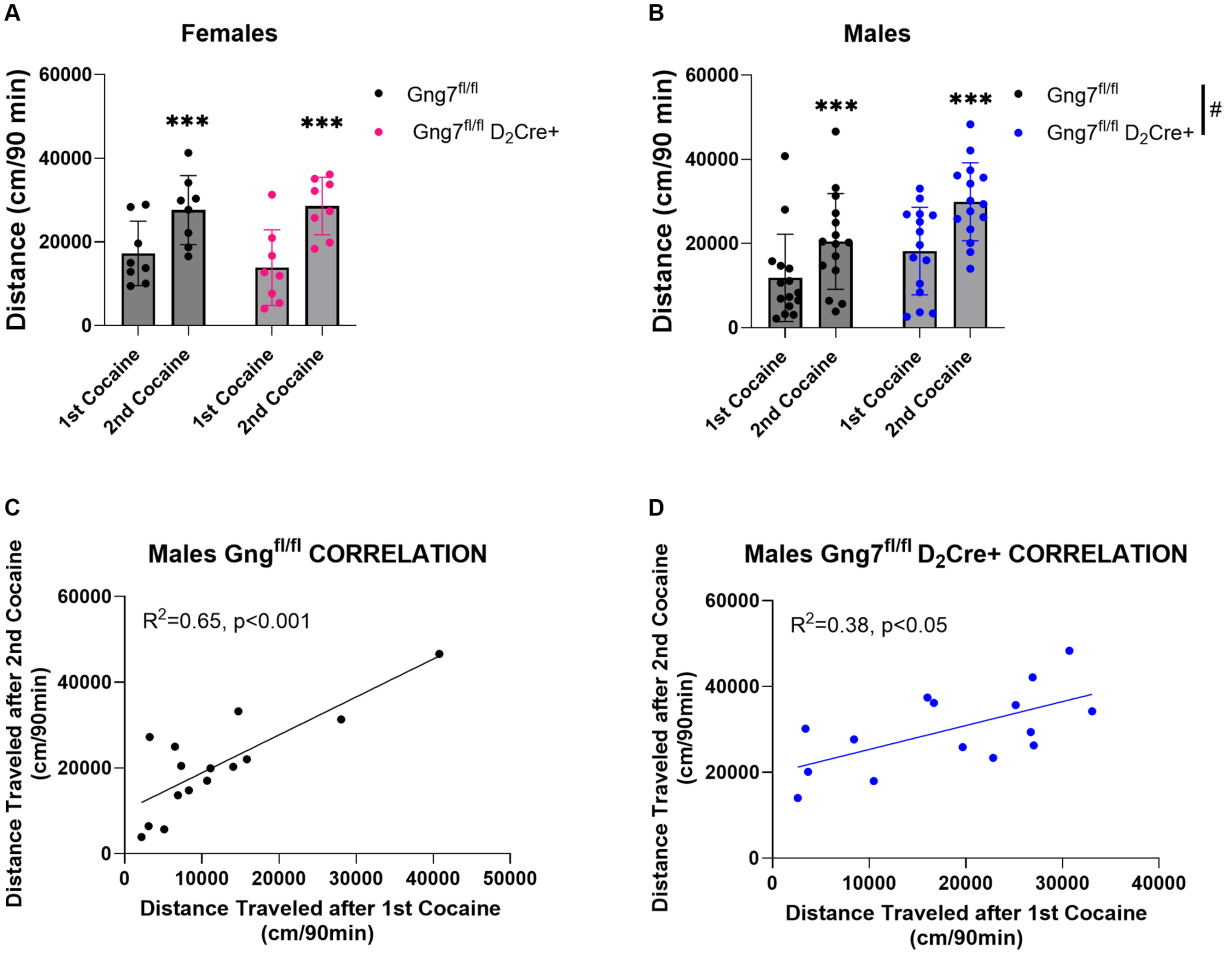
Locomotor sensitization induced by cocaine in *Gng7^fl/fl^* D_2_Cre + and *Gng7^fl/fl^* mice. Open field locomotor activity of *Gng7^fl/fl^* and *Gng7^fl/fl^* D_2_Cre + mice following two cocaine injections. Locomotor responses were measured during 90 min after two cocaine injections administered 1 week apart. **(A)** Female Gng7^fl/fl^ and Gng7^fl/fl^ D_2_Cre + mice showed significant locomotor sensitization to cocaine during the second cocaine exposure. **(B)** Male Gng7^fl/fl^ and Gng7^fl/fl^ D_2_Cre + mice showed significant locomotor sensitization to cocaine, with the latter displaying significant increase in cocaine-induced locomotion. **(C,D)** Correlation analysis depicting the acute (*x* axis) and sensitized locomotor (*y* axis) responses to cocaine in Gng7^fl/fl^ and Gng7^fl/fl^ D_2_Cre + male mice. Both genotypes showed a positive correlation between the responses during the first and second cocaine administration, indicative of cocaine sensitization. Values are expressed as mean ± SEM. Asterisks indicate a statistically significant (**A,B** Repeated measures two-way ANOVA; ***p < 0.001 difference from first injection; #*p* < 0.05 genotype difference, see results for details). *n* = 8–15 mice/group.

In parallel studies, D_2_R-conditional knockout mice were used to examine the specific contribution of the Gα_olf_β_2_γ_7_ heterotrimer in A_2_R signaling on behavioral sensitization to cocaine. Confirming our previous observations (Brunori et al., 2021), male *Gng7*^fl/fl^ D_2_Cre + mice conditional knockout mice displayed significantly higher basal locomotor activity (Supplementary Figure S6). Female *Gng7*^fl/fl^ D_2_Cre + displayed similar acute and sensitized locomotor responses to cocaine as their *Gng7*^fl/fl^ control littermates. The repeated measures two-way ANOVA revealed a sensitization effect [*F*(1,14) = 27.5 *p <* 0.001] but no significant genotype effect [*F*(1,14) = 0.14, ns] or interaction [*F*(1,14) = 0.84, ns] (Figure 9A and Supplementary Figure S6).

Male *Gng7^fl/fl^* D2Cre + mice displayed significantly higher locomotor responses to cocaine independently of the number of injections (Supplementary Figure S6). Repeated measures two-way ANOVA showed a sensitization effect [*F*(1,28) = 51.5, *p* < 0.001] and genotype effect [*F*(1,28) = 5.03, *p* < 0.05] but no interaction between the two factors [*F*(1,28) = 1.17, ns, Figure 9B]. Positive correlations between acute and sensitized responses were observed in both male *Gng7^fl/fl^* D2Cre + and *Gng7^fl/fl^* littermates (Figures 9C,D). These results reveal an important contribution for A_2a_R signaling through the Gα_olf_β_2_γ_7_ heterotrimer in locomotor responses to cocaine. This effect is sex-specific, being observed only in male mice.

Taken together, the combined results from the two different *Gng7* conditional knockout lines uncover circuit-specific roles for the Gα_olf_β_2_γ_7_ heterotrimer in mediating behavioral sensitization to cocaine.

## Discussion

A coordinated balance between the D_1_R and A_2a_R signaling pathways within the two MSN subpopulations making up the striatum is essential for proper control of motor and reward processes (Schwindinger et al., 2003, 2010; Brunori et al., 2021). Notably, dysfunction in one or the other signaling pathway has been associated with various disease states. Our current study sheds light on how regional diversity of *Gng7* mRNA transcripts can be leveraged to impact γ_7_ protein localization and G_olf_ signaling functions in two neurocircuits contributing to addictive disorders. Understanding these regulatory mechanisms is vital for developing targeted interventions.

### Transcriptional regulation of G_olf_ heterotrimer

Our previous work highlighted the importance of the Gαolfβ2γ7 heterotrimer in striatum (Schwindinger et al., 2003, 2010; Liu and Wang, 2019). However, the mechanisms governing the specificity of heterotrimer assembly remain unclear. During the second postnatal week, the striatal expression of both *Gng7* and *Gnal* genes encoding the γ_7_ and α_olf_ subunits increases dramatically. Their coordinated expression may involve specific subsets of transcription factors, such as Gsx2, Dlx1/2, Sp8/9, Isl1, that are required for induction and differentiation of distinct MSN subtypes in this brain region (Cirnaru et al., 2021). Future investigations will explore the *cis-*regulatory elements and factors contributing to their temporal co-expression (Meer et al., 2012; Leppek et al., 2018; Garcia et al., 2022).

### Post-transcriptional regulation of G_olf_ assembly

While the expression of individual α_olf_, β_2_, and γ_7_ subunits within the striatum is coordinately regulated at the transcriptional level, the functionality of the Gα_olf_β_2_γ_7_ complex is dependent on a defined 1:1:1 stoichiometry of the three subunits. A growing body of evidence indicates the proper stoichiometry is achieved by controlling the amount of the γ_7_ protein (Schwindinger et al., 2010), However, the underlying mechanism(s) has not been identified.

Medium spiny neurons (MSNs) express multiple γ subtypes (Schwindinger et al., 2010). Thus, the failure of other γ subtypes to substitute for the γ_7_ protein has been puzzling. Our new findings suggest that the γ_7_ subtype plays a crucial role in driving the assembly of the G_olf_ heterotrimer in specific subcellular compartments, such as dendrites (Huang and Li, 2009b). Of the various *Gng7* isoforms, Transcript 1 is the predominate form in MSNs and is notable for its extended 3’UTR. Similar to other brain mRNAs subject to post-transcriptional regulation and dendritic targeting (Robishaw and Berlot, 2004; Tushev et al., 2018; Mayr, 2019; Yim et al., 2019), we show that Transcript 1 is not efficiently translated into protein and is enriched in the neuropil.

Focusing attention on its extended 3’UTR, there is extensive literature documenting miRNAs that suppress gene expression by binding to the 3′ UTRs of their target mRNAs and acting at the level of translational repression or degradation (Huang and Li, 2009a; Jovičić et al., 2013; Hu et al., 2014; Orang et al., 2014; Mayr, 2019). In the case of Transcript 1, the 3’UTR contains many predicted miRNA binding sites. Of particular interest, we functionally validated two such factors: mir-6972-5p and miR-504-3p. While there is limited information about these regulatory factors, miR-504-3p is expressed in striatum (Zhang et al., 2013) and has been indirectly linked to the γ_7_ and the α_olf_ protein through its upstream D_1_R receptor. Importantly, miR-504 has been shown to bind to the 3’ UTR culminating in allele-specific expression of *DRD1* (Huang and Li, 2009a). Additionally, miR-504-3p has also been directly linked to *Gng7* expression in the hippocampus in response to chronic alcohol administration (Choi et al., 2020) and has been associated with nicotine (Huang and Li, 2009b; Dreyer, 2010), and cocaine dependence (Chen et al., 2013), suggesting a possible role in addictive disorders. Furthermore, mir-504 expression is significantly associated with depressive behavior (Zhang et al., 2013), which is a comorbidity of substance abuse (Calarco and Lobo, 2021). Interestingly, mir-504 has been linked to a cocaine-inducible circRNA, which serves as sponge for miR-504 (Bu et al., 2019), thereby providing a possible mechanism for increasing localized γ_7_ production and G_olf_ assembly within dendritic spines. Finally, studies have shown that miR-504 is regulated by estrogen (Feng et al., 2020), providing a possible explanation for how miR-504-mediated modulation of γ_7_ expression plays a role in the sex influences on cocaine sensitization. Altogether, our results, along with existing literature, strongly support a role of miR-504-mediated regulation of γ_7_ protein under pathophysiological conditions including cocaine sensitization. While other miRNAs have been linked to *Gng7* expression in various contexts, including miR-423-5p (Martinez and Peplow, 2017), miR-326-3p (Carrick et al., 2016), miR-212-5p (Hollander et al., 2010), miR-326-3p, and mir-135-5p (Chen, 2020), none of these miRNAs were observed to alter *Gng7* expression in our experimental system.

### Dendritic morphology of MSNs

Local translation of synaptic proteins ensures high-fidelity of signal transmission and is critical for neuronal maturation and synaptic plasticity (Kang and Schuman, 1996; Huber et al., 2000; Swanger and Bassell, 2013; Schanzenbächer et al., 2016; Scarnati et al., 2018; Bae and Miura, 2020). Here we show that deletion of γ_7_ results in significant changes in dendritic spine density and size on MSNs from adult mice. Specifically, we observed a significantly higher density of dendritic spines in both male and female *Gng7*^−/−^ mice compared to wildtype littermates, along with a significant reduction in spine head diameter in females. Previous studies identified Gα_s_-cAMP signaling as a key regulator of MSN synaptogenesis during early postnatal striatal development (Shen et al., 2008). Although we did not characterize dendritic spines based on their morphology or MSN subtype localization, our results extend these findings by supporting a role for the Gα_olf_β_2_γ_7_ -cAMP signaling in the pruning and/or maturation of spines. Based on evidence that Gα_olf_ replaces Gα_s_ during postnatal development of the striatum (Rius et al., 1994), we speculate that Gα_olf_-cAMP signaling within dendritic spines serves as an important mediator for activity-dependent refinement of striatal plasticity into adulthood (Bian et al., 2015). To confirm this hypothesis, future studies will extend the analysis of dendritic spines in *Gng7*^−/−^ mice at different developmental time points and will investigate the impact of Gα_olf_β_2_γ_7_-cAMP signal loss on MSN synaptic plasticity.

### Functional implications

Alterations in dendritic spine morphology is a common feature of several neuropsychiatric disorders, including addiction (Penzes et al., 2011; Spiga et al., 2014). For example, changes in dendritic spine density on MSNs associated with repeated psychostimulant exposure have been suggested to underlie long-lasting changes in synaptic plasticity, which contribute to persistent drug-seeking behaviors (Robinson and Kolb, 1997; Robinson, 1999; Shen et al., 2009; Dumitriu et al., 2012). Previously, we demonstrated that global γ_7_ deletion in mice affects acute locomotor responses to certain psychostimulant drugs (Schwindinger et al., 2003, 2010; Brunori et al., 2021). Here, we show that global deletion of γ_7_ in mice did not alter acute cocaine locomotor response or cocaine-induced behavioral sensitization. However, using conditional D_1_R- and D_2_R-conditional knock-out mice, we observed distinct roles of Gα_olf_β_2_γ_7_ heterotrimer signaling in mediating cocaine behavioral responses.

Despite several reports pointing to a critical role of Gα_olf_ signal downstream D_1_R receptor for acute responses to psychostimulants (Corvol et al., 2007; Biever et al., 2017), we demonstrate that D_1_R signaling via the specific Gα_olf_β_2_γ_7_ heterotrimer does not affect acute responses to either amphetamine (Brunori et al., 2021) or cocaine but seems to be a critical component in the establishment of cocaine sensitization. While this discrepancy is likely related to differences in the genetic model (*Gng7^fl/fl^* D_1_Cre + vs. *Gnal*^+/−^) and in the sensitization protocol (2-injection protocol vs. 5 consecutive days of cocaine) used in our study, we cannot rule out the possibility that Gα_olf_ forms alternative heterotrimeric complexes with γ subunits other than γ_7_ to signal downstream D_1_R and influence acute psychostimulant response. Nevertheless, our results confirm that D_1_R-MSNs are a critical substrate for cocaine-induced behavioral sensitization (Karlsson et al., 2008; Valjent et al., 2009; Hikida et al., 2010) and suggest that D_1_R signaling through Gα_olf_β_2_γ_7_ heterotrimer is implicated in this process. Specifically, we observe that cocaine sensitization failed to be established in γ_7_-D_1_R knockout mice characterized by high acute cocaine behavioral responsiveness. Previous studies in outbred rats have characterized a similar animal model based on individual differences in initial locomotor response to cocaine and identified distinct behavioral and biochemical features in high vs. low cocaine responders, including lack of sensitization and reduced dopamine clearance after cocaine challenge, without evident changes in postsynaptic D_1_R or D_2_R receptors (Sabeti et al., 2002; Gulley et al., 2003; Sabeti et al., 2003). Furthermore, there is evidence that production of dendritic spines after cocaine sensitization preferentially occurs in neurons that are more strongly activated by cocaine (Koya et al., 2012), whereas lack of new spine formation affects the expression of locomotor sensitization (Brown et al., 2011; Cahill et al., 2018; Cai et al., 2022). Based on this information, we speculate that cocaine sensitization in high cocaine responder mice may reliably depend on dopamine-dependent mechanisms and may selectively engage D_1_R-Gα_olf_β_2_γ_7_ heterotrimer signal to generate the associated plasticity on dendritic spines. The lack of genotype effects in females could be explained by sex-dependent differences in extracellular dopamine levels after the cocaine challenge (Griffin and Middaugh, 2006) and accordingly sensitization in both females and low cocaine responder males may preferentially rely on extra striatal areas and/or glutamate transmission (Kelley et al., 2003; Kalivas et al., 2005). Understanding how Gα_olf_β_2_γ_7_ heterotrimer loss impacts cocaine-mediated alterations in MSN synaptic function remains an interesting question for future studies.

In agreement with previous observations (Brunori et al., 2021), we found that elimination of Gα_olf_β_2_γ_7_ heterotrimer signal downstream of A_2A_Rs in D_2_R-MSNs results in increased sensitivity to the locomotor-stimulating effects of cocaine. However, at the dose of cocaine tested (20 mg/kg), this effect was only observed in male mice. While mechanisms underlying this sex-dependent effect are still being investigated, we hypothesize that ovarian influences on both pre-synaptic dopamine signaling (Calipari et al., 2017; Becker and Chartoff, 2019) as well as post-synaptic D2R receptor function (Bazzett and Becker, 1994) may play a role. However, when mice were challenged with a second dose of cocaine both females and males showed similar sensitization. Our findings are in line with previous studies demonstrating that inhibition of postsynaptic A_2A_Rs is associated with increased locomotor responses to cocaine, due to its permissive effect on D_2_R activity (Ferre et al., 2008) but has no effect on the expression of locomotor sensitization (Filip et al., 2006; Haynes et al., 2019).

## Summary

In conclusion, our findings demonstrate the importance of subcellular localization of γ7 protein for directing Gα_olf_β_2_γ_7_ heterotrimer site-specific assembly and downstream signaling, shaping dendritic morphology of MSNs, and modulating responses to drugs of abuse. Notably, miRNAs are increasingly emerging as novel targets in the pathophysiology of drug addiction. Among a large panel of miRs expressed in brain, we show that miR-504 downregulates reporter luciferase activity and decreases *Gng7* expression by targeting its 3’UTR. Because γ_7_ protein is the rate limiting step in the assembly of the functional G_olf_ heterotrimer and subsequent downstream signaling, this miRNA-mediated modulation of *Gng7* expression is predicted to alter the amount of the γ_7_ protein, and hence, the level of functional G_olf_ heterotrimer in the direct and indirect pathways in the brain. Since G_olf_ heterotrimer is a critical mediator of dopamine and adenosine actions, the post-transcriptional regulation of Gng7 expression is also predicted to contribute to the molecular mechanisms underlying cocaine sensitization. Interestingly, miR-504 has been associated with alcohol, nicotine, and cocaine dependence (Huang and Li, 2009b; Dreyer, 2010; Chen et al., 2013; Choi et al., 2020). The mechanism(s) remain to be elucidated, but the observed dendritic changes in *Gng7* knock-out mice suggest that G_olf_ heterotrimer could contribute to altered structural plasticity, and/or signaling imbalances between D_1_R and D_2_R neurocircuitry implicated in functional plasticity. Collectively, our results support the role for specific γ subunits in the assembly of highly specialized G protein complexes that are critical for proper brain development and function. Understanding how these complexes are regulated and assembled may lead to new opportunities for therapeutic intervention of several disease states including addiction.

## Data availability statement

The original contributions presented in the study are included in the article/Supplementary material, further inquiries can be directed to the corresponding author.

## Ethics statement

The animal study was approved by Florida Atlantic University IACUC. The study was conducted in accordance with the local legislation and institutional requirements.

## Author contributions

OP: Conceptualization, Data curation, Formal analysis, Investigation, Methodology, Validation, Visualization, Writing – original draft, Writing – review & editing. GB: Conceptualization, Data curation, Formal analysis, Investigation, Methodology, Project administration, Supervision, Validation, Visualization, Writing – review & editing. YW: Conceptualization, Data curation, Formal analysis, Investigation, Methodology, Project administration, Supervision, Validation, Visualization, Writing – review & editing. JR: Conceptualization, Funding acquisition, Investigation, Project administration, Resources, Supervision, Validation, Visualization, Writing – review & editing.
