# Supplementary material for: Post-transcriptional regulation and subcellular localization of G-protein γ7 subunit: implications for striatal function and behavioral responses to cocaine
Source: Front Neuroanat. 2024 May 2;18:1394659. doi: 10.3389/fnana.2024.1394659 (PMC11100332; doi:10.3389/fnana.2024.1394659)
Supplement: Supplementary file 1 [file Data_Sheet_1.PDF]

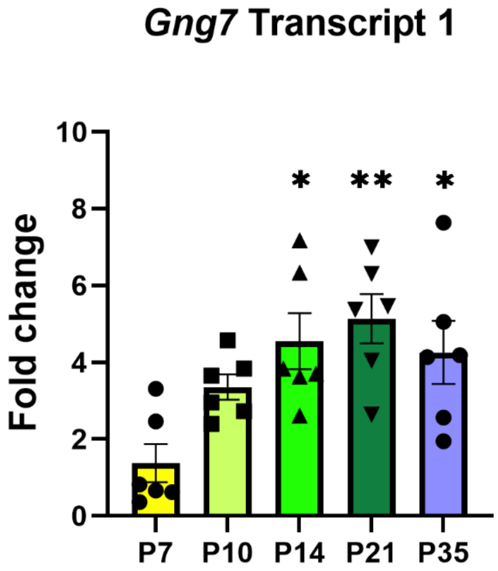

Supplementary Figure 1. The postnatal developmental *Gng7* Transcript 1 mRNA levels in *Gng7*$^{+/+}$ mouse striatum. *Gng7* transcript 1 expression in the striatum at five developmental time points (P= postnatal day). Values are expressed as mean ± SEM. Asterisks indicate a statistically significant difference from the P7 time point (one-way ANOVA; *$p < 0.05$, **$p < 0.01$). n=6 mice/time point

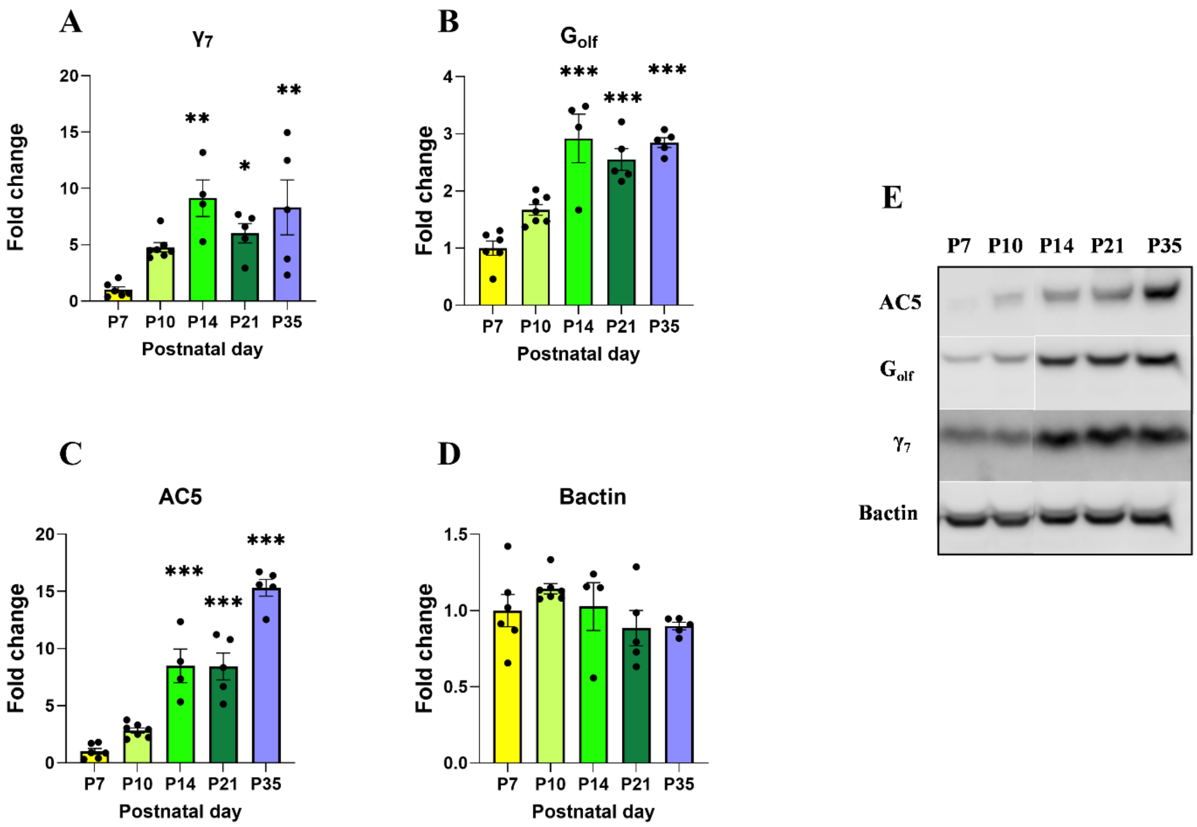

Supplementary Figure 2. The postnatal developmental γ_7, G_{olf}, and AC5 protein levels in *Gng7*$^{+/+}$ mouse striatum. A) γ_7, **B)** G_{olf} **C)** AC5 and **D)** Beta-actin protein expression in the striatum at five developmental time points (P= postnatal day). Each column represents a normalized ratio (fold-change) relative to total protein loading and to P7. **E)** Representative blots. Values are expressed as mean ± SEM. Asterisks indicate a statistically significant difference from the P7 time point (one-way ANOVA; $^{**}p < 0.01$, $^{***}p < 0.001$). n=5-7 mice/time point

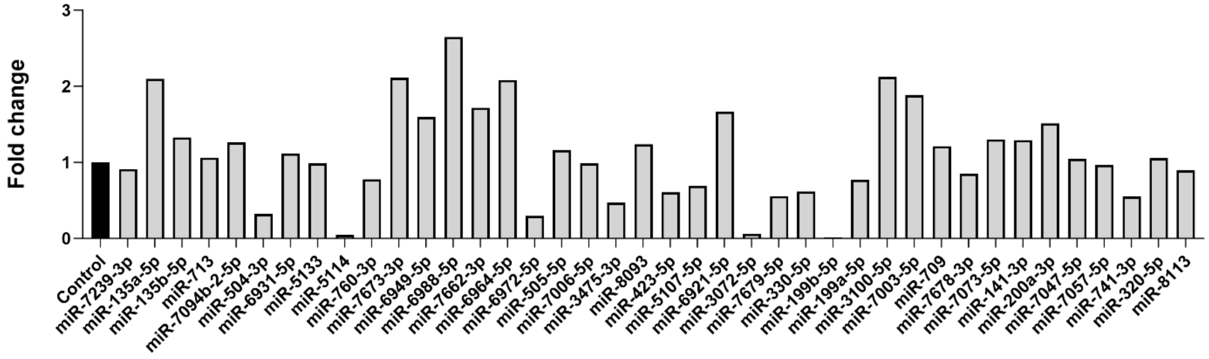

Supplementary Figure 3. Screening of individual miRNA for post-transcriptional regulation of *Gng7*. Gene expression of *Gng7* following co-transfection of *Gng7* vector with selected miRNAs in HEK 293 cells as assessed by qPCR.

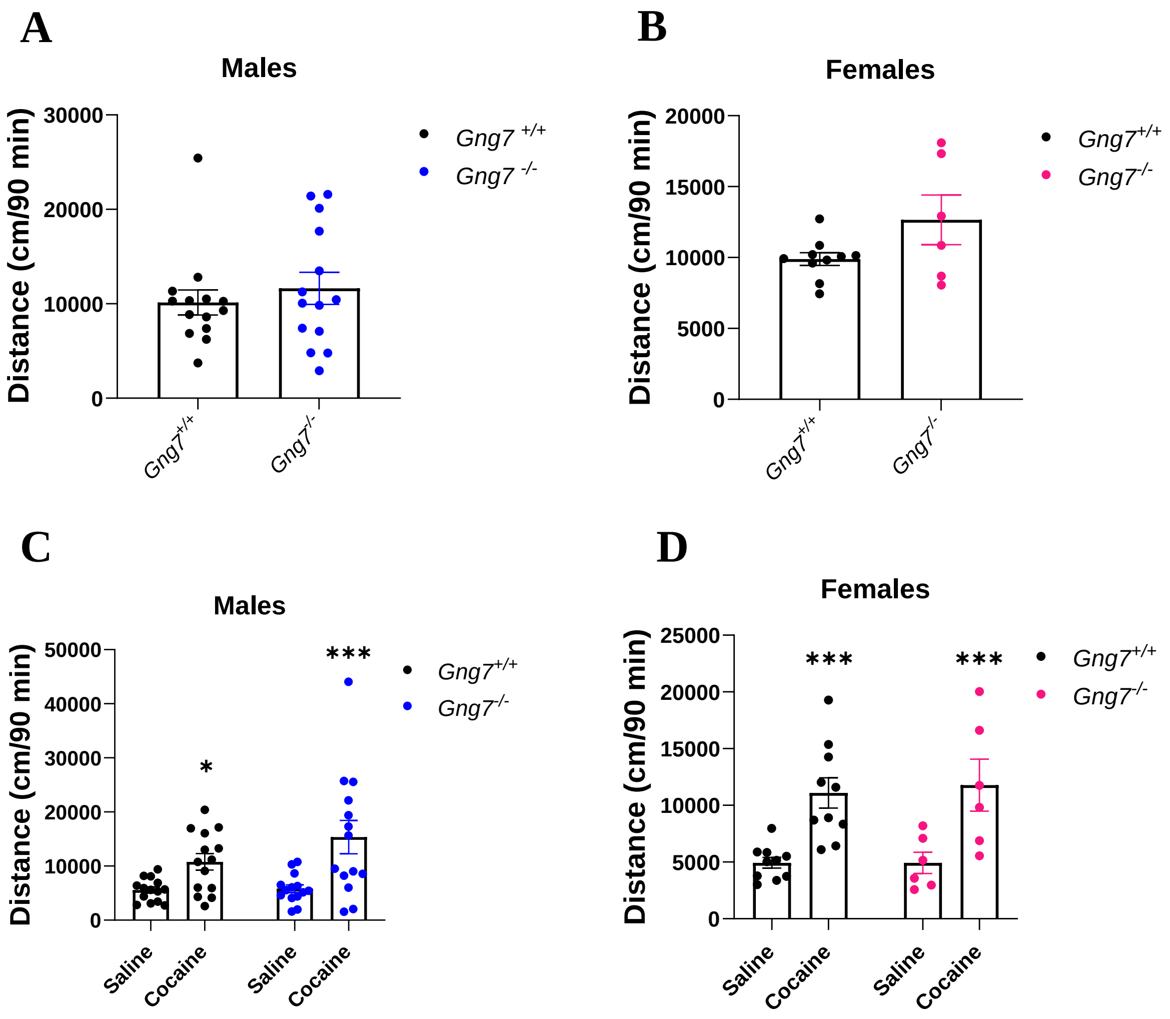

Supplementary Figure 4. Spontaneous locomotion and locomotion induced by cocaine of *Gng7*$^{+/+}$ and *Gng7*$^{-/-}$ mice. Open field locomotor activity of *Gng7*$^{+/+}$ and *Gng7*$^{-/-}$ mice. Both **A)** male and **B)** female *Gng7*$^{+/+}$ and *Gng7*$^{-/-}$ mice showed a normal spontaneous locomotor response. Locomotor responses were measured for 90 min after saline and cocaine were administered. Both **C)** male and **D)** female *Gng7*$^{+/+}$ and *Gng7*$^{-/-}$ mice showed significant locomotor to cocaine. Values are expressed as mean ± SEM. (F Repeated measures two-way ANOVA, ***p < 0.001 difference from saline). n=6-14 mice/sex

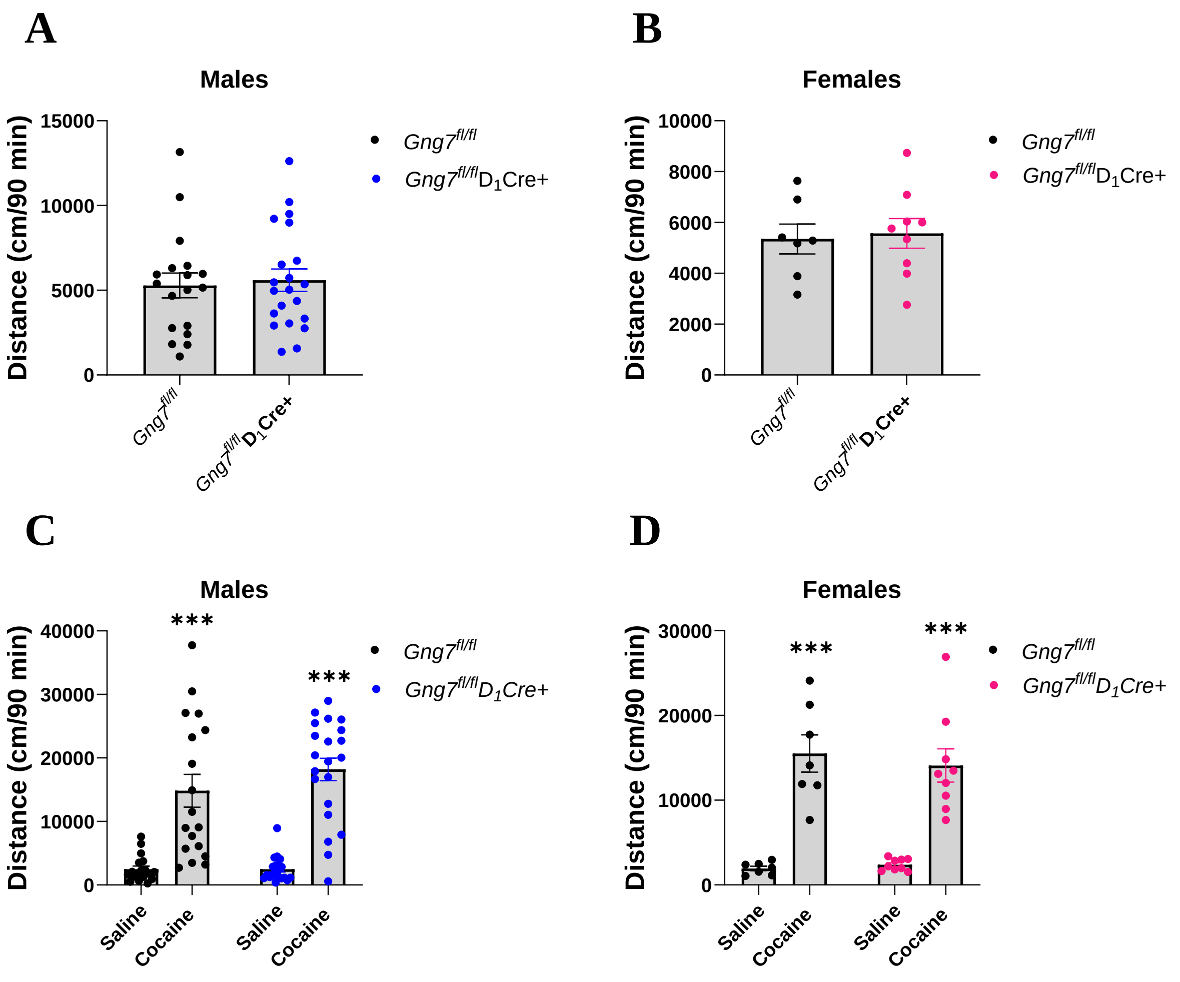

Supplementary Figure 5 Spontaneous locomotion and locomotion induced by cocaine of *Gng7*$^{fl/fl}$ D$_1$Cre+ and *Gng7*$^{fl/fl}$ mice. Open field locomotor activity of *Gng7*$^{fl/fl}$ and *Gng7*$^{fl/fl}$ D$_1$Cre+. Both **A)** male and **B)** female *Gng7*$^{fl/fl}$ and *Gng7*$^{fl/fl}$ D$_1$Cre+ mice showed a normal spontaneous locomotor response. Locomotor responses were measured for 90 min after saline and cocaine were administered. Both **C)** male and **D)** female *Gng7*$^{fl/fl}$ and *Gng7*$^{fl/fl}$ D$_1$Cre+ mice showed significant locomotor to cocaine. Values are expressed as mean ± SEM. (F Repeated measures two-way ANOVA, ***p < 0.001 difference from saline). n=7-21 mice/sex

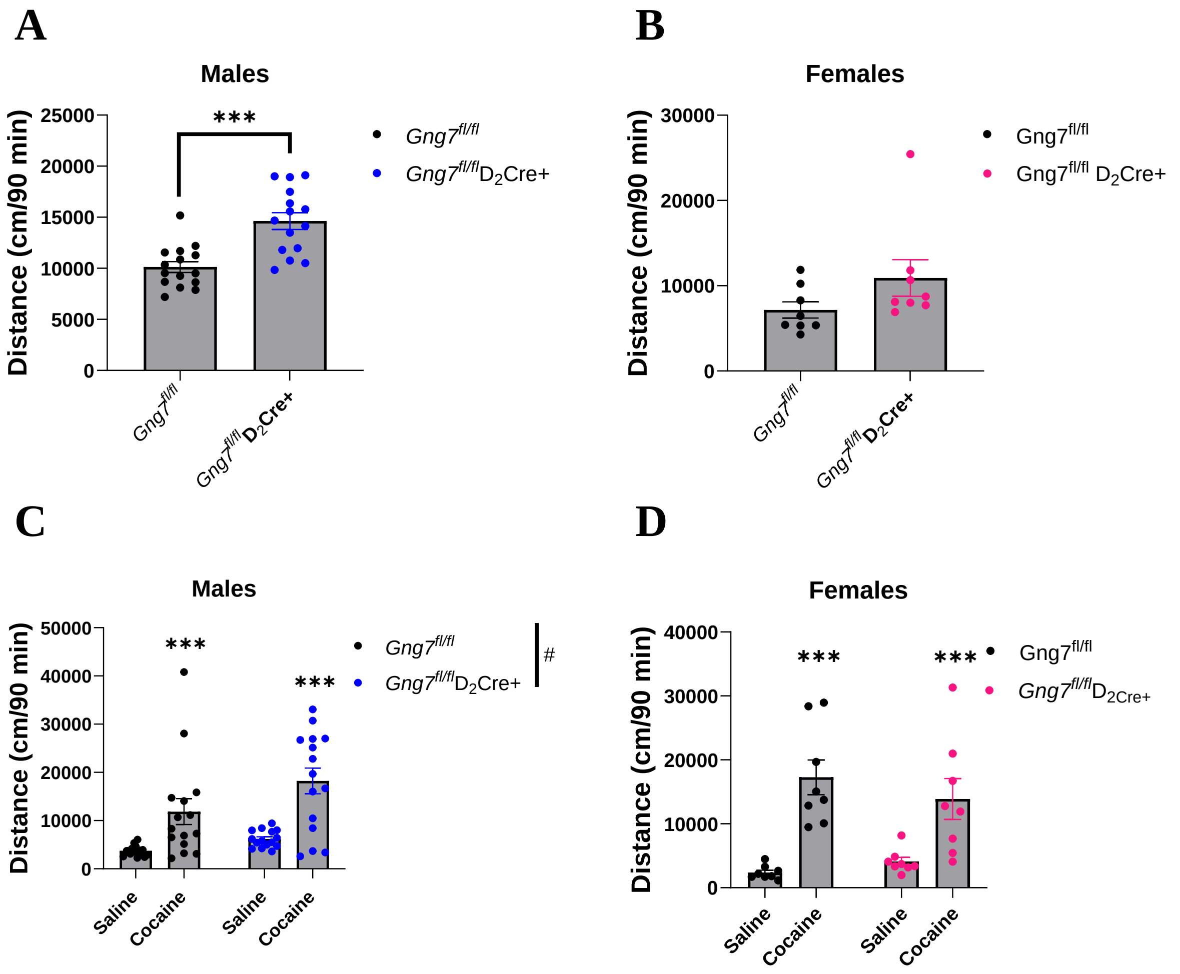

Supplementary Figure 6 Spontaneous locomotion and locomotion induced by cocaine of *$Gng7^{fl/fl}$* D_2Cre+ and *$Gng7^{fl/fl}$* mice. Open field locomotor activity of *$Gng7^{fl/fl}$* and *$Gng7^{fl/fl}$* D_2Cre+. **A)** Male *$Gng7^{fl/fl}$* D_2Cre+ mice showed a significant increase in spontaneous locomotion compared to male *$Gng7^{fl/fl}$* mice (Student's t-test ***$p < 0.001$) **B)** Female *$Gng7^{fl/fl}$* and *$Gng7^{fl/fl}$* D_2Cre+ mice showed a normal spontaneous locomotor response. Locomotor responses were measured for 90 min after saline and cocaine were administered. Both **C)** male and **D)** female *$Gng7^{fl/fl}$* and *$Gng7^{fl/fl}$* D_2Cre+ mice showed significant locomotor to cocaine. Values are expressed as mean ± SEM. (F Repeated measures two-way ANOVA, ***$p < 0.001$ difference from saline, Genotype difference #$p<0.05$). n=8-15 mice/sex

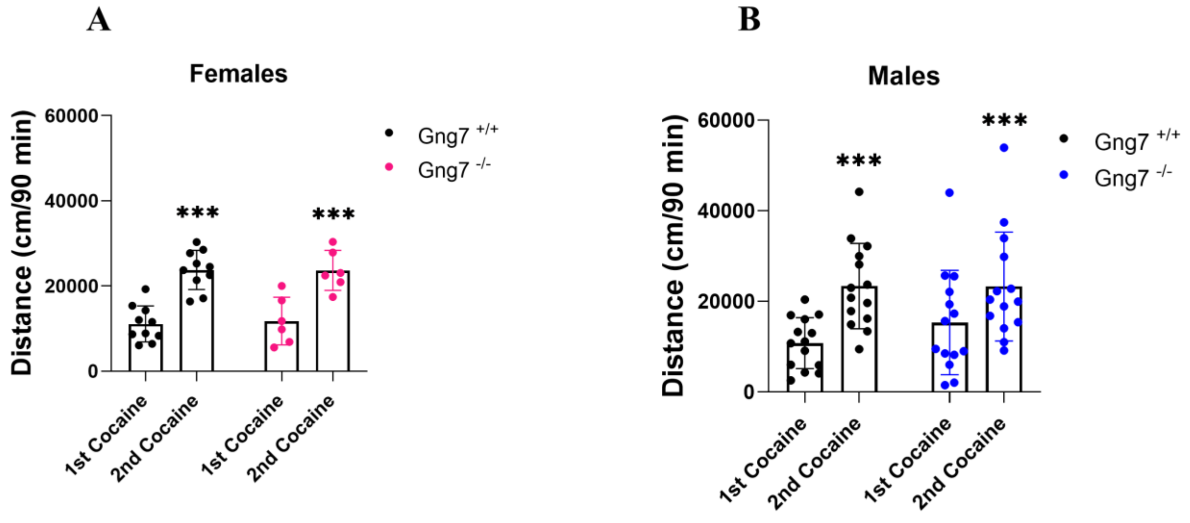

Supplementary Figure 7. Locomotor sensitization induced by cocaine of *Gng7*$^{+/+}$ and *Gng7*$^{-/-}$ mice. Open field locomotor activity of *Gng7*$^{+/+}$ and *Gng7*$^{-/-}$ mice following two cocaine injections. Locomotor responses were measured for 90 min after two cocaine injections were administered one week apart. Both **A)** female and **B)** male *Gng7*$^{+/+}$ and *Gng7*$^{-/-}$ mice showed significant locomotor sensitization to cocaine. Values are expressed as mean ± SEM. (F Repeated measures two-way ANOVA, ***$p < 0.001$ difference from the first injection). n=6-14 mice/sex
